# The CXCL9/SPP1 polarity axis in tumor-associated macrophages: immunoregulatory and prognostic significance in non-small cell lung cancer

**DOI:** 10.3389/fimmu.2026.1763652

**Published:** 2026-04-17

**Authors:** Houqiang Li, Miaoyan Liu, Shenghan Xu, Yike Zhou, Youlang Zhou, Jiahai Shi, Lou Zhong

**Affiliations:** 1 Department of Thoracic Surgery, Affiliated Hospital of Nantong University, Nantong, China; 2 Research Center of Clinical Medicine, Affiliated Hospital of Nantong University, Nantong, China

**Keywords:** *CXCL9/SPP1* polarity axis, macrophage polarization, non-small cell lung cancer (NSCLC), prognosis, tumor microenvironment (TME), tumor-associated macrophages (TAMs)

## Abstract

This study addresses the limitations of the traditional M1/M2 binary classification for tumor-associated macrophages (TAMs) in non-small cell lung cancer (NSCLC) by introducing a NSCLC-specific functional framework based on the *CXCL9/SPP1* (CS) expression ratio. Through the integration of single-cell and bulk transcriptomic data, the research identified four distinct TAM subpopulations. Among these, the *CXCL9^+^SPP1^−^* subpopulation exhibited macrophages with anti-tumor features, whereas the *CXCL9^−^SPP1^+^* subpopulation showed macrophages with pro-tumor features. A robust CS-polarity-associated tumor microenvironment (TME) six-gene signature was constructed and validated using extensive machine-learning optimization. This model effectively stratified NSCLC patients into high-risk and low-risk groups, with high-risk patients displaying an immunosuppressive TME enriched in M0/M2 macrophages. The study further demonstrated the dynamic plasticity of TAM polarity through pseudotime trajectory analysis and validated key gene expression. For the first time, this study introduces the *CXCL9/SPP1* polarity axis into the field of non-small cell lung cancer (NSCLC). By integrating single-cell trajectory analysis, we reveal the dynamic differentiation patterns of TAM polarity in NSCLC. Furthermore, utilizing a combination of 101 machine learning algorithms, we constructed the first six-gene prognostic model based on this polarity axis, achieving precise risk stratification for NSCLC patients and enabling correlative analysis of the immune status within the tumor microenvironment.

## Introduction

Lung cancer remains the leading cause of cancer-related incidence and mortality globally, with an estimated 2.48 million new cases and 1.81 million deaths in 2022 (1). Non-small cell lung cancer (NSCLC) accounts for more than 85% of all lung cancer cases, encompassing major subtypes such as lung adenocarcinoma and lung squamous cell carcinoma (2). Despite recent breakthroughs in targeted therapy and immunotherapy, the overall prognosis for NSCLC remains poor due to tumor heterogeneity, therapeutic resistance, and the lack of accurate prognostic systems (3, 4). Therefore, in-depth exploration of the molecular mechanisms underlying the occurrence and progression of NSCLC, identification of prognostic biomarkers with clinical value, and construction of precise prediction models are of great clinical significance for optimizing patient risk stratification and guiding individualized treatment.

The tumor microenvironment (TME) serves as the “soil” that supports the survival and progression of tumor cells, and its compositional complexity and dynamic remodeling capacity directly affect the tumor behavior (5). Tumor-associated macrophages (TAMs), the most abundant population of immune cells in the TME, account for 30%-50% of the total tumor-infiltrating immune cells and function as key regulators of tumor progression (6). TAMs exhibit significant functional heterogeneity. The traditional view classifies them into the classically activated M1 (anti-tumor phenotype) and alternatively activated M2 (pro-tumor phenotype): M1 macrophages activate adaptive immune responses by secreting cytokines such as interleukin-12 (IL-12) and interferon-γ (IFN-γ), thereby directly killing tumor cells; in contrast, M2 macrophages secrete immunosuppressive factors including interleukin-10 (IL-10) and transforming growth factor-β (TGF-β), which promote tumor angiogenesis, invasion, metastasis, and immune escape (7). However, accumulating evidence indicates that the phenotype of TAMs does not conform to a simple binary classification, but rather exists in a continuous functional polarization spectrum. Regulated dynamically by tumor cells, stromal cells, and cytokine networks, TAM functional complexity is inadequately captured by their functional complexity (8). Therefore, identifying novel biomarkers that accurately distinguish the functional subtypes of TAMs and exploring their regulatory networks have become key research priorities in tumor immunology in recent years.

C-X-C motif chemokine ligand 9 (*CXCL9*) and secreted phosphoprotein 1 (*SPP1*) are two cytokines with distinct functions that play crucial roles in tumor immune regulation and have recently been closely associated with TAM polarization in recent years (9, 10). CXCL9, a member of the CXC chemokine family induced by IFN-γ, is primarily secreted by macrophages and dendritic cells. By binding to the C-X-C motif chemokine receptor 3 (CXCR3), CXCL9 specifically recruits effector immune cells such as CD8^+^ T cells and natural killer (NK) cells to infiltrate tumor tissues, thereby enhancing anti-tumor immune responses (11). Existing studies have shown that high CXCL9 expression correlates with favorable prognosis across multiple tumor types and may serve as a potential biomarker for radiotherapy-induced immune activation (9). *SPP1* (also known as osteopontin)is a secreted glycophosphoprotein widely involved in cell adhesion, migration, inflammatory response, and immune regulation (12). In the TME, SPP1 is mainly secreted by TAMs, tumor cells, and stromal cells. By binding to integrin receptors (e.g., αvβ3, αvβ5) and cluster of differentiation 44 (*CD44*), SPP1 activates signaling pathways such as signal transducer and activator of transcription 3 (*STAT3*) and nuclear factor-kappa B (*NF-κB*), thereby promoting tumor cell proliferation, invasion, and angiogenesis (13). In addition, SPP1 induces macrophage polarization toward the M2 phenotype, suppresses effector T cells, and reinforces an immunosuppressive microenvironment (14). In NSCLC, high SPP1 expression is positively associated with TNM stage and lymph node metastasis, and it serves as an independent risk factor for poor prognosis (15).

Recently, emerging evidence across multiple solid tumors has proposed the *CXCL9/SPP1* (CS) polarity axis as a functional framework that replaces the traditional M1/M2 dichotomy (16). In these contexts, *CXCL9*^+^ TAMs typically shape an anti-tumor, inflammatory microenvironment by recruiting CD8^+^ T cells, whereas *SPP1*^+^ TAMs exert immunosuppressive and pro-tumorigenic effects by promoting angiogenesis, matrix remodeling, and CD8^+^ T cell dysfunction. This dual mechanism has been extensively validated across various cancers, including head and neck squamous cell carcinoma, hepatocellular carcinoma, and clear cell renal cell carcinoma (17–19). However, despite its critical immunoregulatory value across multiple cancers, specific investigations into the CS polarity axis within the highly heterogeneous tumor microenvironment of non-small cell lung cancer (NSCLC) remain absent. Furthermore, most existing studies are limited to cross-sectional phenotypic characterizations, and lack in-depth exploration of the dynamic differentiation trajectories and polarization plasticity of these TAM subpopulations (20). Crucially, no study to date has integrated single-cell transcriptomics, multi-omics data, and large-scale machine learning algorithms to systematically explore the prognostic value and regulatory networks of this polarity axis, nor has such an approach been translated into a robust, clinically applicable prognostic model. Therefore, this study aims to systematically investigate this polarity axis in NSCLC to address this critical research gap.

To bridge this research gap, this study aims to systematically investigate the expression patterns and functional significance of *CXCL9* and *SPP1* in NSCLC TAMs, screen prognostic genes associated with this polarity, and construct a robust risk prediction model by integrating single-cell transcriptomic data, bulk transcriptomic data, and clinical information. The specific objectives are as follows: to clarify the expression characteristics and polarization correlation of CXCL9 and SPP1 in NSCLC TAMs; to screen *CXCL9/SPP1*-related differentially expressed genes (DEGs) through multi-omics data integration and identify core prognostic genes; to construct a stable prognostic risk model using 101 combinations of machine learning algorithms and evaluate its predictive efficacy in independent cohorts; to analyze the correlations of the prognostic model with clinical features, immune microenvironment, tumor mutations, and immune checkpoint expression; and to reveal the regulatory networks of *CXCL9/SPP1*-polarized TAMs at the single-cell level, including transcription factors, metabolic pathways, and cell communication mechanisms. Through multi-dimensional bioinformatics analysis, this study is expected to provide a novel prognostic evaluation tool for NSCLC patients, identify key regulatory targets of TAM polarization, and offer a theoretical basis for developing precise immunotherapeutic strategies.

## Materials and methods

### Data preparation

Transcriptomic data and clinical information of TCGA-NSCLC were downloaded from The Cancer Genome Atlas (TCGA) database (https://portal.gdc.cancer.gov/) as the training set. The GSE50081 (GPL570) dataset was retrieved from the Gene Expression Omnibus (GEO) database (https://www.ncbi.nlm.nih.gov/geo/) as the validation set. The GSE135222 (GPL16791) advanced NSCLC cohort with PD-1/PD-L1 inhibitor treatment information was used as an external validation set. In addition, the GSE198099 (GPL24676) dataset was also downloaded from GEO for single-cell analysis. E-MTAB-13530, downloaded from the BioStudies database (https://www.ebi.ac.uk/biostudies/), was used for spatial transcriptome analysis.

### Single-cell RNA sequencing data analysis

Single-cell RNA sequencing data from the GSE198099 dataset were processed using the Seurat package. Low-quality cells were filtered based on standard quality control criteria, and the remaining data were normalized, followed by identification of the top 2,000 highly variable genes. Principal component analysis (PCA) was performed for dimensionality reduction, and cells were clustered using a resolution of 0.2. Cell subtypes were annotated according to canonical marker genes.

Fisher’s exact test was applied to identify differential cell populations, followed by functional enrichment analysis using ReactomeGSA. The expression patterns of *CXCL9* and *SPP1* were visualized, and the Wilcoxon test was used to compare their expression levels between tumor and control groups (P < 0.05). Cells showing significant differential expression of both genes were defined as key cells, of which macrophages were identified as the key cell subset.

Macrophages were further subjected to re−dimensionality reduction, reclustering, and subtype annotation. Differentially expressed genes1 (DEGs1) among macrophage polarization states in the tumor group were identified using the FindMarkers function with thresholds of |average log2 fold change (log2FC)| > 0.5 and adjusted P-value (p.adjust) < 0.05. Detailed information was provided in the **Supplementary Material**.

### Spatial transcriptome analysis

Spatial transcriptome data were processed and analyzed using the Seurat and spacexr packages following standardized bioinformatic workflows. Briefly, basic quality control and data normalization were conducted after metadata annotation; multi-sample integration, dimensionality reduction, clustering and differential marker gene screening were then performed sequentially, followed by visualization of marker gene spatial expression profiles.

Cell type deconvolution was implemented via the spacexr package, with related results and annotated Seurat objects preserved. Key cell spots were extracted and classified into four subtypes based on gene expression thresholds, with subtype distribution quantified across groups. Additionally, neighborhood composition of annotated cells was analyzed, and Wilcoxon test along with paired sample difference analysis were performed for statistical verification.

Spatial transcriptomic analyses of CXCL9 and SPP1 in NSCLC were performed based on the E-MTAB-13530 dataset. Briefly, the annotated spatial transcriptomic data were processed using the Seurat package, and the expression levels of CXCL9 and SPP1 were extracted for subsequent analyses. Cell subset enrichment, spatial correlation and co-localization patterns were assessed via appropriate statistical tests, including Fisher’s exact test, Spearman correlation analysis and Moran’s I tests. Multiple testing correction was conducted using the Benjamini-Hochberg method to control the false discovery rate (FDR). Spatial distribution, aggregation and mutual exclusivity of CXCL9 and SPP1 were visualized using tailored plotting parameters.

Detailed bioinformatic procedures, including specific function parameters and analytical thresholds, are provided in the **Supplementary Materials**.

### Differential expression analysis and differential analysis of CXCL9 and SPP1 high/low expression groups

The DESeq2 package was used to identify DEGs by comparing tumor and control groups in the TCGA dataset (|log2FC| > 0.5 and p.adjust < 0.05), resulting in DEGs2.

In tumor samples from the TCGA training set, samples were divided into high and low expression groups based on the optimal cutoff value of CXCL9 (or SPP1) gene expression. The Wilcoxon rank-sum test was used to analyze the expression differences of CXCL9 (or SPP1) between the two groups. The log-rank test was performed using the R package “survival” to assess the survival difference between high and low expression groups, and Kaplan-Meier (K-M) survival curves were plotted using the R package “survminer”. DEGs between CXCL9 (or SPP1) high and low expression groups were analyzed using the DESeq2 package (|log2FC| > 0.5 and p.adjust < 0.05). The union of DEGs from the two groups was obtained after deduplication to generate DEGs3. Detailed information was provided in the **Supplementary Material**.

### Gene set enrichment analysis

GSEA was performed to explore the potential signaling pathways and related biological functions of CXCL9 and SPP1 in NSCLC. Differential analysis between CXCL9 (or SPP1) high and low expression groups was conducted using the R package “DESeq2” to calculate log2FC values, which were sorted in descending order for GSEA. GSEA was performed using the “clusterProfiler” package and the “c2.cp.kegg.v7.4.symbols.gmt” background set from the Molecular Signatures Database (MSigDB) (https://www.gsea-msigdb.org) (p.adjust < 0.05, |normalized enrichment score (NES)| > 1).

Additionally, Spearman correlation analysis between CXCL9/SPP1 and all other genes was performed using the R package “psych” to obtain correlation coefficients. Genes were ranked by correlation coefficients in descending order to generate gene ranking lists for GSEA. GSEA was also conducted using the “clusterProfiler” package and the “c2.cp.kegg.v2023.1.Hs.symbols.gmt” background set from MSigDB (p.adjust < 0.05, |NES| > 1).

### Acquisition of intersection genes and screening of prognostic genes

To obtain common genes, the intersection of DEGs1, DEGs2, and DEGs3 was calculated using the R package “ggvenn” to generate intersection genes. Univariate Cox regression analysis was performed on these intersection genes to screen genes with hazard ratio (HR) ≠ 1 and P < 0.1. Prognostic genes that further met the proportional hazards (PH) assumption (P > 0.05) were retained for in-depth analysis. A prediction model with 101 algorithm combinations was constructed using a leave-one-out cross-validation (LOOCV) framework based on ten machine learning methods: stepwise Cox regression (R package “survival”), Random Survival Forest (RSF, R package “randomForestSRC”), Elastic Net (Enet, R package “glmnet”), Supervised Principal Components (SuperPC, R package “superpc”), Partial Least Squares Regression for Cox Models (plsRcox, R package “plsRcox”), CoxBoost (R package “CoxBoost”), Survival Support Vector Machine (survival-SVM, R package “survivalsvm”), Lasso (R package “glmnet”), Ridge (R package “glmnet”), and Gradient Boosting Machine (GBM, R package “gbm”). The model with the optimal concordance index (C-index) was selected.

### Construction and validation of prognostic risk model

Tumor samples in the TCGA training set and GSE50081 validation set were divided into high-risk and low-risk groups based on the median risk score calculated by the optimal model among the 101 machine learning algorithms. The distribution of samples and risk scores, sorted by patient risk score from low to high, was visualized. K-M curves were plotted using the R package “survminer”, and survival status plots were generated to evaluate the OS between the two groups. Receiver operating characteristic (ROC) curves for 2-, 3-, and 5-year survival were plotted using the R package “timeROC”, and the area under the curve (AUC) was calculated to assess the predictive accuracy of the risk model (AUC > 0.6). Heatmaps showing the expression levels of prognostic genes in high- and low-risk groups were generated.

### Correlation analysis between risk score and clinical features

To analyze the relationship between different clinicopathological features and high/low risk groups, chi-square tests (P < 0.05) were used to explore the associations between meaningful clinicopathological features and risk groups based on patient data with clinical information in the TCGA training set. The distribution of high- and low-risk patients across different clinical features was analyzed to evaluate the correlation between risk groups and clinical characteristics.

### Immune infiltration analysis

To investigate the infiltration distribution of immune cells in high- and low-risk groups, the R package “CIBERSORT” and the standard immune cell expression file “LM22.txt” were obtained from CIBERSORT (https://cibersort.stanford.edu/). The “CIBERSORT” package was used to analyze the gene expression matrix data of high-risk and low-risk groups in the TCGA training set, and the relative proportions of immune cells in all samples were visualized. Spearman correlation analysis was performed using the R package “psych” to calculate the correlations between immune cells with significant differences and between prognostic genes and differential immune-infiltrating cells(|correlation coefficient (r)| > 0.3 and P < 0.05).

### GSEA of high- and low-risk groups

GSEA was conducted to explore the functional enrichment of high- and low-risk groups. Differential analysis between high- and low-risk groups was performed using the R package “DESeq2” to calculate log2FC values, which were sorted in descending order for GSEA. GSEA was performed using the “clusterProfiler” package and the “c2.cp.kegg.v7.4.symbols.gmt” background set from MSigDB (p.adjust < 0.05, |NES| > 1).

### Tumor purity analysis

To further explore the differences in tumor scores between high- and low-risk groups, the ESTIMATE algorithm from the ESTIMATE database (https://bioinformatics.mdanderson.org/estimate/) was used to calculate the stromal score, immune score, and ESTIMATE score (comprehensive score of stromal and immune scores) for samples in the TCGA training set. The Wilcoxon rank-sum test was used to compare the differences in stromal score, immune score, and ESTIMATE score between high- and low-risk groups (P < 0.05).

### Tumor mutation analysis

Gene mutations are key factors affecting biological evolution and frequently occur in cancers. Exploring gene mutations is of great significance for disease screening and treatment. Based on somatic mutation data of NSCLC samples in the TCGA training set, the top 20 most frequently mutated genes in high- and low-risk groups were visualized using the “oncoplot” function in the R package “maftools”. The “mafCompare” function was used to analyze genes in MAF files of the two cohorts and identify those mutated in at least 5 samples. Differentially mutated genes were identified using the “DESeq2” package with thresholds of p.adjust < 0.05 and |log2FC| > 1. Univariate Cox analysis was performed using the “survival” package, and genes with HR ≠ 1 and P < 0.05 were retained. After further PH assumption testing, genes with P > 0.05 were defined as key mutated genes. Additionally, mutation analysis of key prognostic genes was performed using NSCLC patient data from the cBioPortal database (https://www.cbioportal.org/).

### Immune checkpoint analysis and immunotherapy analysis

To analyze the expression differences of immune checkpoint genes between high- and low-risk groups, the Wilcoxon rank-sum test was used to compare the expression levels of 67 immune checkpoint genes between the two groups (P < 0.05). The results of this analysis provide insights into the potential role of immune checkpoint-related genes in prognostic prediction and may offer new targets for precise immunotherapy in high-risk patients.

In the GSE135222 cohort, patients with progression-free survival (PFS) > 180 days and no progression were defined as responders, whereas the remaining patients were defined as non−responders. A Cox proportional hazards model was constructed based on 101 machine−learning feature genes, and the risk score was calculated. Samples were divided into high− and low−risk groups according to the median risk score. The difference in treatment response rates between the two groups was compared using Fisher’s exact test. The PFS differences and prognostic value between different risk groups were analyzed by the log-rank test and Cox regression analysis.

### Pseudotime analysis

Bubble plots and violin plots were generated to show the expression of prognostic genes across all cell types.The R package Monocle can arrange cells in a simulated temporal order to display cellular developmental trajectories, such as cell differentiation. To understand the differentiation process of key cell subpopulations and the expression patterns of prognostic genes at different differentiation stages of key cell subpopulations, the annotated key cell subpopulations were arranged along the corresponding cell trajectory. Using the Monocle package, key cell subpopulations were classified into different differentiation states based on gene expression profiles to complete the pseudotime analysis. Dynamic heatmaps were plotted to observe the dynamic changes of prognostic genes.

### Cell communication analysis

Cell communication refers to the process where signals from one cell are transmitted to another cell through mediators, triggering corresponding responses. Intercellular communication via chemical signaling molecules is the most common communication method in animals and plants. Ligand-receptor complex-mediated cell-cell communication plays a crucial role in coordinating various biological processes such as development, differentiation, and inflammation. To analyze the communication between all cells, the R package CellChat was used to perform cell communication analysis in tumor and control samples from the single-cell dataset GSE198099, calculate potential ligand-receptor interactions, and obtain interaction signals between different cell types. CellPhoneDB is a curated database of ligands, receptors, and their interactions. With CellChatDB.mouse as the reference, CellChat was used to explore the cell-cell interactions of annotated cells.

### Single-cell transcription factor analysis

Transcription factors (TFs) control various cellular processes and states by regulating gene programs. Single-Cell Regulatory Network Inference and Clustering (SCENIC) analysis aims to identify shared regulatory networks using putative regulatory binding sites in promoter regions and can guide the identification of TFs and cell states. The GENIC3 method was used to infer co-expression modules between TFs and candidate target genes. Each module, referred to as a regulon, consists of a TF and its target genes. The cisTarget human motif database was used to enrich gene signatures, and signature genes were filtered according to default procedures. To comprehensively understand the TFs and their controlled programs in the sequenced macrophage subpopulations, the R package “SCENIC” was used to analyze the enrichment of TF-bound genes in each subpopulation. The activity of each regulon in each cell (regulon specificity score, RSS) was calculated by AUCell in the R package SCENIC. The top five specific regulons in different key cells were identified based on RSS, and the results were visualized.

### Metabolic pathway analysis

To understand the metabolic status, functions, and potential regulatory mechanisms of key cells, metabolic activity analysis was performed. The VISION algorithm in the R package “scMetabolism” enables quantitative analysis of metabolic activity at single-cell resolution. Based on macrophage subtypes, their single-cell expression matrices were extracted. The metabolic activity of 85 Kyoto Encyclopedia of Genes and Genomes (KEGG) pathways was evaluated using scMetabolism, and each cluster was scored using the VISION algorithm. Finally, the activity score of each cluster in each metabolic pathway was obtained based on the standard single-cell matrix file.

### Protein validation using the human protein atlas

To evaluate prognostic gene expression at the protein level, the HPA database (https://www.proteinatlas.org/) was used to analyze their expression in NSCLC and normal tissues. HPA classifies protein expression levels into four grades: “High”, “Medium”, “Low”, and “Not detected”.

### Correlation analysis between *CXCL9/SPP1* and prognostic genes

Spearman correlation analysis from the R package “psych” was used to analyze the expression correlations between prognostic genes and *CXCL9/SPP1*. Correlation analysis was performed between the expression levels of prognostic genes and *CXCL9/SPP1*, respectively. Screening and statistical analysis were conducted (correlation coefficient (cor)| > 0.3 and P < 0.05).

### Cell culture

All cell lines were obtained from the American Type Culture Collection (ATCC). H1299 cells were cultured in RPMI-1640 medium (Cytiva) supplemented with 10% fetal bovine serum. BEAS-2B and A549 cells were cultured in DMEM (Fuheng Biology) supplemented with 10% fetal bovine serum (Gibco). The mouse macrophage cell line RAW 264.7 was cultured in DMEM high-glucose basal medium (Cytiva) supplemented with 10% FBS and 1% penicillin-streptomycin. All the cells were maintained in a humidified environment at 37 °C with 5% CO2 (Thermo).

### Macrophage polarization induction

For polarization induction, RAW 264.7 cells were seeded into six-well plates at a density of 3 × 10^5^ cells/well and cultured overnight. After washing once with PBS, the medium was replaced with DMEM containing for six hours of serum starvation. Subsequently, the cells were treated as follows: M0 control group: DMEM. M1 polarization group: DMEM supplemented with lipopolysaccharide (LPS, 100 ng/mL, Sigma-Aldrich) and recombinant mouse interferon-γ (IFN-γ, 20 ng/mL, PeproTech). M2 polarization group: DMEM supplemented with recombinant mouse interleukin-4 (IL-4, 20 ng/mL, PeproTech) and recombinant mouse interleukin-13 (IL-13, 20 ng/mL, PeproTech). All groups were incubated for 24 hours. Each experiment was performed with three biological replicates.

### Validation of prognostic signatures by quantitative real-time PCR

Total RNA was isolated from samples using TRIzol reagent (Invitrogen, USA), and the integrity and purity of the extracted RNA were verified prior to subsequent experiments. The first-strand cDNA was synthesized via reverse transcription using the HiScript II RT SuperMix kit (Vazyme, China), following the manufacturer’s recommended protocol to ensure efficient reverse transcription. Quantitative Real-Time PCR (qRT-PCR) reactions were conducted on a QuantStudio six Real-Time PCR System (Thermo Fisher Scientific, USA) using the SYBR Green Master Mix (Vazyme, China) as the fluorescent detection reagent. Each sample was assayed in triplicate to ensure the reproducibility and reliability of the experimental data. Relative expression was calculated by the 2^(–ΔΔCt) method with GAPDH as reference. Primer information was presented in the **Supplementary Material**.

### Multiplex immunofluorescence staining

Perform multiple immunofluorescence (mIF) staining on 3 pairs of human lung adenocarcinoma and normal tissue sections. This study strictly adhered to ethical guidelines and was approved by the Ethics Committee of Nantong University Affiliated Hospital (Approval Number: 2021-L142). and all participating patients signed written informed consent forms. Paraffin-embedded tissue sections were dewaxed, rehydrated, and subjected to heat-induced antigen retrieval in EDTA buffer (pH 8.0) at 100 °C for 15 min. Endogenous peroxidase activity was blocked by treatment with 3% hydrogen peroxide. After serum blocking, three sequential rounds of antibody incubation and TSA signal amplification were performed. In the first round, sections were incubated with rabbit anti-human CXCL9 monoclonal antibody (1:500, Servicebio, Cat# 22355-1-AP), followed by HRP-conjugated goat anti-rabbit secondary antibody (ready-to-use, Servicebio, Cat# G1302), and signal was developed using iF488-Tyramide (1:500, Servicebio, Cat# G1231). After antibody stripping, the second round was performed using rabbit anti-human SPP1 monoclonal antibody (1:5000, Servicebio, Cat# GB11500) with HRP-conjugated goat anti-rabbit secondary antibody, followed by iF647-Tyramide (1:500, Servicebio, Cat# G1232). Following another stripping step, the third round was performed using mouse anti-human CD68 monoclonal antibody (1:50000, Servicebio, Cat# GB153150) with HRP-conjugated goat anti-mouse secondary antibody (ready-to-use, Servicebio, Cat# G1301), and signal was developed using iF555-Tyramide (1:500, Servicebio, Cat# G1233). All primary antibody incubations were performed overnight at 4 °C, while secondary antibody and TSA incubations were carried out at room temperature under light-protected conditions. After completing the staining procedure, sections were counterstained with DAPI for nuclear visualization, treated with an autofluorescence quencher to reduce background, and mounted with an anti-fade mounting medium.

### Statistical analysis

All data processing, statistical analysis, and plotting were performed in GraphPad Prism (version 10) and R (version 4.3.1). Student’s t-test or two-way ANOVA was used to compare the differences between groups, as appropriate. The results for continuous variables were presented as the mean ± standard deviation. A P value ≤0.05 was considered statistically significant. For plot presentation, ns indicates p > 0.05, * indicates p < 0.05, ** indicates p < 0.01, *** indicates p < 0.001, and **** indicates p < 0.0001.

## Results

### Single-cell transcriptomic analysis reveals cellular composition differences between tumor and normal tissues in NSCLC

**Figure 1:**
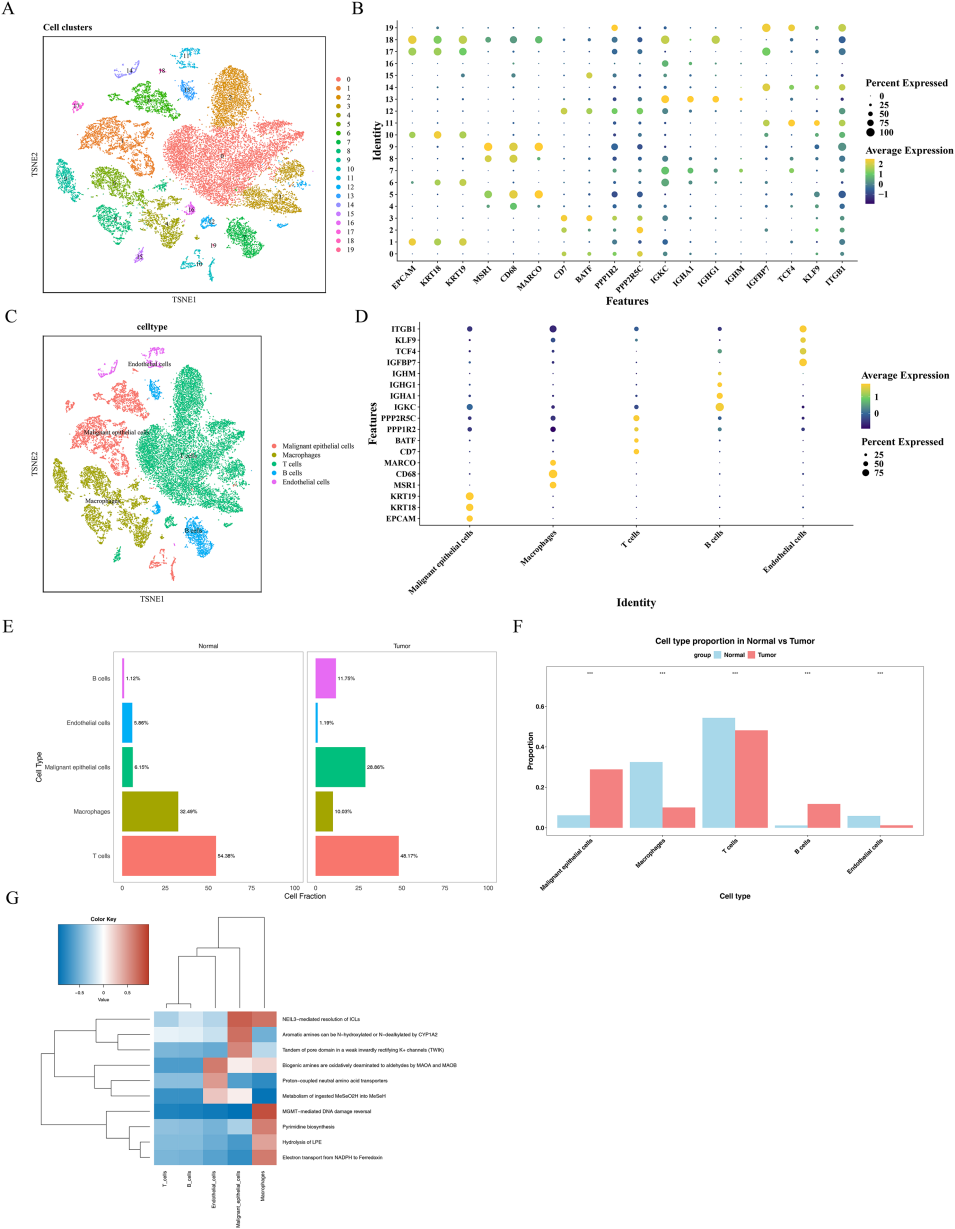
Screening of single-cell data. **(A)** Cells were clustered into 20 clusters after dimensionality reduction. **(B)** A bubble plot was generated to show the highly expressed marker genes in each cluster. **(C)** A total of five cell types were identified after cell annotation. **(D)** A bubble plot was constructed to display the highly expressed marker genes in the annotated cell types. **(E)** T cells were found to account for a relatively high proportion. **(F)** A total of five cell types showing significantly different proportions were identified. **(G)** Functional enrichment analysis was performed on the five cell types.

scRNA-seq analysis was performed on tumor and paired adjacent normal tissue samples from the GSE198099 dataset. After quality control, 27,161 high-quality cells and 24,471 genes were retained for subsequent analyses. Following data normalization, 2,000 highly variable genes were identified. Cell clustering was performed based on the top 30 significant principal components (PCs), resulting in the cells into 20 subclusters (**Figure 1A**, **Supplementary Figure 1**). Using known cell markers (21), we annotated five major cell types: malignant epithelial cells, macrophages, T cells, B cells, and endothelial cells (**Figure 1B**, **Supplementary Table 1**). t-distributed Stochastic Neighbor Embedding (t-SNE) plots were used to visualize the annotated cell types (**Figure 1C**). Marker gene expression exhibited high specificity across cell clusters, with distinct expression patterns (**Figure 1D**). Macrophages, endothelial cells, and T cells were more abundant in normal tissues, whereas malignant epithelial cells and B cells were enriched in tumor tissues (**Figure 1E**). Fisher’s exact test confirmed significant differences in cell type distribution between tumor and normal tissues, with macrophages, T cells, and endothelial cells enriched in normal tissues, and malignant epithelial cells and B cells enriched in tumor tissues (**Figure 1F**). We systematically analyzed the significantly enriched signaling pathways in each cell cluster. The top 10 most significantly enriched pathways were primarily involved in DNA damage repair (e.g., “MGMT-mediated DNA damage reversal” and “NEIL3-mediated resolution of ICLs”), metabolic processes (e.g., “Biogenic amines are oxidatively deaminated to aldehydes by MAOA and MAOB”, “Metabolism of ingested MeSeO2H into MeSeH”, and “Pyrimidine biosynthesis”), and ion and molecular transport (e.g., “Proton-coupled neutral amino acid transporters” and “Tandem of pore domain in a weak inwardly rectifying K+ channels (TWIK)”) (**Figure 1G**).

### Identification of macrophage subpopulations and analysis of differentially expressed genes

**Figure 2:**
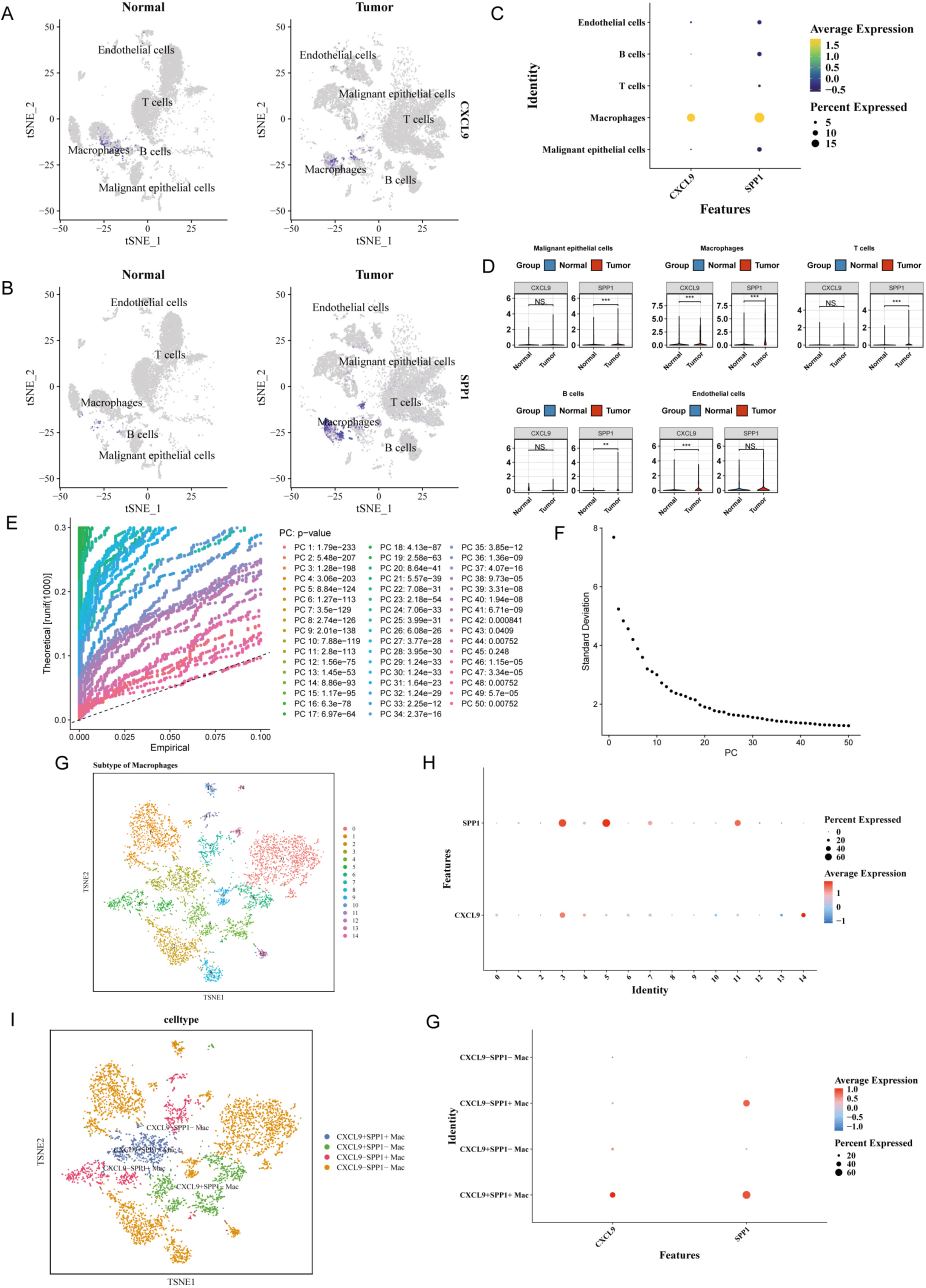
Identification of key cells and annotation of macrophage subsets. **(A, B)** t-SNE plots showed that CXCL9 and SPP1 were distributed mainly in macrophages among the differential cells. **(C)** The localization of CXCL9 and SPP1 genes in different cell types was determined, and macrophages accounted for a high proportion. **(D)** The expression of CXCL9 and SPP1 in differential cells was analyzed, and both genes were differentially expressed in macrophages. **(E, F)** The identification of 30 PCs was performed. **(G)** Macrophages were clustered into 15 subclusters after dimensionality reduction. **(H)** A bubble plot was used to show the expression of CXCL9 and SPP1 in different cell types; both genes were highly expressed in subcluster 3. **(I)** Macrophage subsets were annotated into 4 subpopulations. **(J)** A bubble plot was generated to display the high expression of CXCL9 and SPP1 in the annotated cells.

To characterize the expression distribution of *CXCL9* and *SPP1* across different cell types and identify key cell populations, we analyzed their expression patterns in tumor and normal samples. t-SNE visualization revealed that *CXCL9* and *SPP1* were predominantly enriched in macrophages, with significantly higher expression in tumor-associated macrophages compared with those from normal tissues (**Figures 2A, B**). Localization analysis further confirmed that both *CXCL9* and *SPP1* exhibited high proportion and average expression levels in macrophages (**Figure 2C**). The Wilcoxon rank-sum test was used to compare the expression differences of *CXCL9* and *SPP1* between tumor and normal tissues across cell types. Cells with significant differential expression of both genes were defined as key cells. This analysis identified macrophages as the primary key cell population, as they showed concurrent differential expression of both *CXCL9* and *SPP1* (**Figure 2D**). Therefore, macrophages were selected as the key cell population for subsequent analyses.

To further dissect the heterogeneity of macrophages within the TME, the annotated macrophage population was subjected to independent analysis. Based on the top 30 statistically significant PCs (**Figures 2E, F**), macrophages were re-clustered into 15 subpopulations (**Figure 2G**). Based on the expression patterns of *CXCL9* and *SPP1*, these subpopulations were categorized into four functionally distinct macrophage groups: *CXCL9*^+^*SPP1*^+^ Mac, *CXCL9*^-^*SPP1*^-^ Mac, *CXCL9*^+^*SPP1*^-^ Mac, and *CXCL9*^-^*SPP1*^+^ Mac (**Figures 2H, I**; **Supplementary Table 2**). Annotation bubble plots confirmed that these marker genes effectively distinguished the subpopulations (**Figure 2G**).

**Figure 3:**
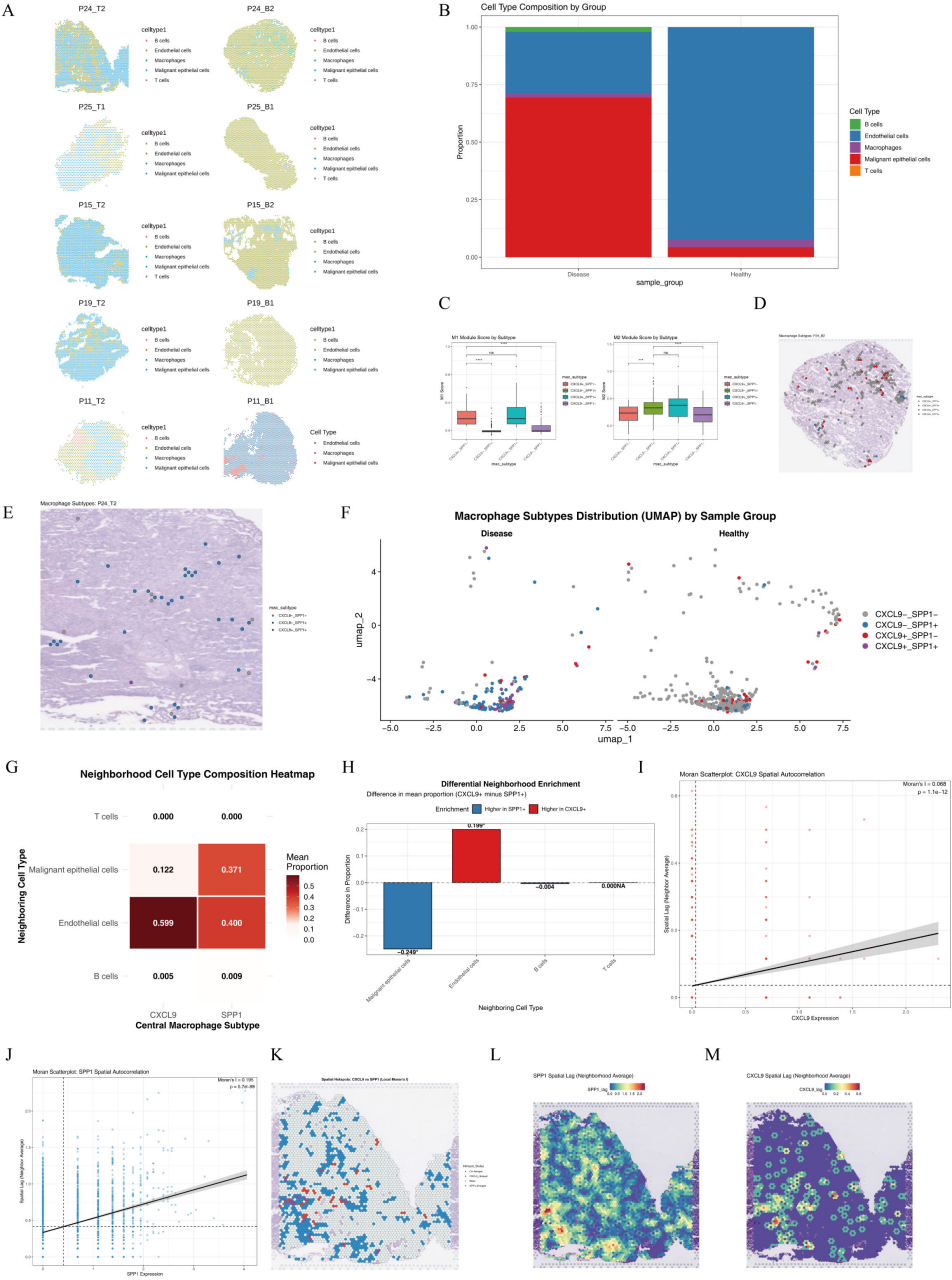
RCTD cell type deconvolution. **(A)** Visualization of spatial distribution of cell types in spatial transcriptomics between disease and healthy groups; **(B)** Cell type composition of spatial transcriptomics between disease and healthy groups; **(C)** Comparative analysis of M1/M2 module scores in four macrophage subtypes (Significance levels were indicated as follows: *p < 0.05, **p < 0.01, ***p < 0.001, ****p < 0.0001, and ns for not statistically significant); Spatial distribution characteristics of macrophage subtypes in adjacent normal tissue (P24_B2, **(D)**] and tumor tissue [P24_T2, **(E)**] of non-small cell lung cancer (NSCLC) patient P24; **(F)** UMAP distribution of macrophage subtypes in NSCLC tumor and paratumor tissues; **(G)** Average proportion of the 8 nearest neighbor cells around the two macrophage subtypes; **(H)** Differences in neighborhood composition between the two macrophage subtypes; **(I)** Spatial lag Moran scatter plot analysis of CXCL9 gene expression; **(J)** Spatial lag Moran scatter plot analysis of SPP1 gene expression; **(K)** CXCL9/SPP1 spatial hotspot distribution map; **(L)** Spatial lag distribution map of SPP1; **(M)** Spatial lag distribution map of CXCL9.

Spatial transcriptome analysis revealed that the diseased and healthy groups were mainly composed of malignant epithelial cells and endothelial cells, respectively, with distinct macrophage abundance between groups (**Figures 3A,B**). Among the four macrophage subsets polarized from M1/M2 phenotypes, significant differences were observed between *CXCL9*^+^*SPP1*^−^ and *CXCL9*^−^*SPP1*^+^ subsets (**Figure 3C**). In NSCLC patient P24, the *CXCL9*^+^*SPP1*^−^ subset was predominantly enriched in high-dimensional spatial regions of the diseased group. Endothelial cells accounted for a significantly higher proportion in the neighborhood of *CXCL9*^+^ cells (0.599) than in *SPP1*^+^ cells (0.400), while malignant epithelial cells were more abundant around *SPP1*^+^ cells (0.371). *SPP1*^+^ cells were highly prevalent in malignant epithelial cells, clearly illustrating the *CXCL9/SPP1* polarization pattern in tumor tissues (**Figures 3D–H**; **Supplementary Figures 2**–**5**).

Global Moran’s I tests were performed. High and statistically significant positive Moran’s I values were observed for both *CXCL9* and *SPP1*, indicating that significant spatial clustering was exhibited by the two genes. Spatial lag scatterplots showed that high expression levels were detected in the neighborhoods of spots with high expression, while low expression levels were found in the neighborhoods of spots with low expression. These results confirmed that distinct spatial regionalization patterns were formed by *CXCL9* and *SPP1*, and that their high-expression regions were spatially independent, allowing stable local functional enrichment zones to be established (**Figures 3H, I**). Spatial hotspot analysis was conducted. The hotspot regions of *CXCL9* and *SPP1* were found to be spatially separated with almost no overlap, and few co-hotspot regions were identified. These findings further verified that mutually exclusive spatial expression patterns were displayed by *CXCL9* and *SPP1*. Spatial lag distribution maps indicated that *SPP1* was predominantly expressed at moderate-to-high levels in clustered regions, with prominent high-value hotspots formed in local areas. In contrast, *CXCL9* was mainly expressed at moderate-to-low levels, with only scattered high-expression clusters observed in limited regions. In summary, significant spatial clustering was exhibited by both *CXCL9* and *SPP1* in NSCLC tissues, and their high-expression regions were spatially independent and mutually exclusive (**Figures 3I–M**).

**Figure 4:**
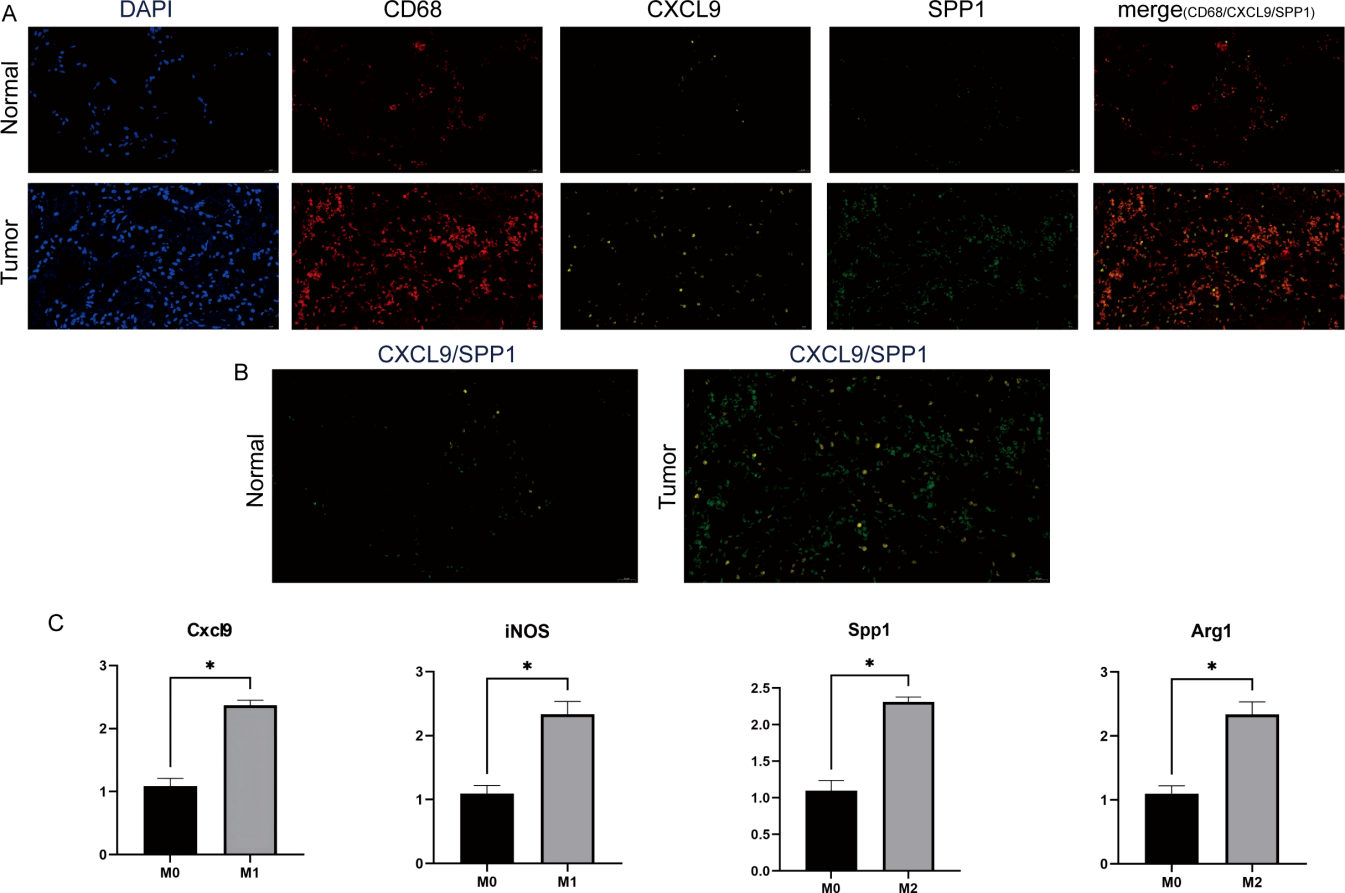
Validation of CXCL9^+^ and SPP1^+^ macrophage expression and polarization characteristics in lung cancer tissues. **(A)** Representative mIF staining for CXCL9+ TAMs and SPP1+ TAMs in lung tissue sections from the normal and LUAD tumor groups. DAPI (blue), CD68 (red), CXCL9 (yellow), SPP1 (green) are shown, along with individual and merged channels. (n = 3 per group). Scale bar, 20 μm. **(B)** Representative mIF staining of CXCL9+ TAMs (yellow) and SPP1+ TAMs (green) in lung tissue sections from the normal and LUAD tumor groups (n = 3 per group). Scale bar, 20 μm. **(C)** qRT-PCR validation of macrophage polarization. The M1 group showed high expression of Cxcl9 and iNOS, while the M2 group showed high expression of Spp1 and Arg1. Asterisks indicate statistically significant differences (*p < 0.05).

mIF staining confirmed that SPP1^+^ macrophages, characterized by the colocalization of CD68 and SPP1 protein expression, and CXCL9^+^ macrophages, characterized by the colocalization of CD68 and CXCL9 protein expression, were enriched in LUAD tumors (**Figures 4A,B**). The qRT-PCR results showed that, compared with the M0 control group, the mRNA expression levels of *Cxcl9* and *iNOS* were significantly upregulated in the M1 polarization group, while the expression levels of *Spp1* and *Arg1* were significantly increased in the M2 polarization group (p < 0.01) (**Figure 4C**). These findings confirm the successful establishment of the macrophage polarization model and demonstrate that *CXCL9* is specifically enriched in M1-like macrophages, whereas *SPP1* is closely associated with M2-like macrophages.

**Figure 5:**
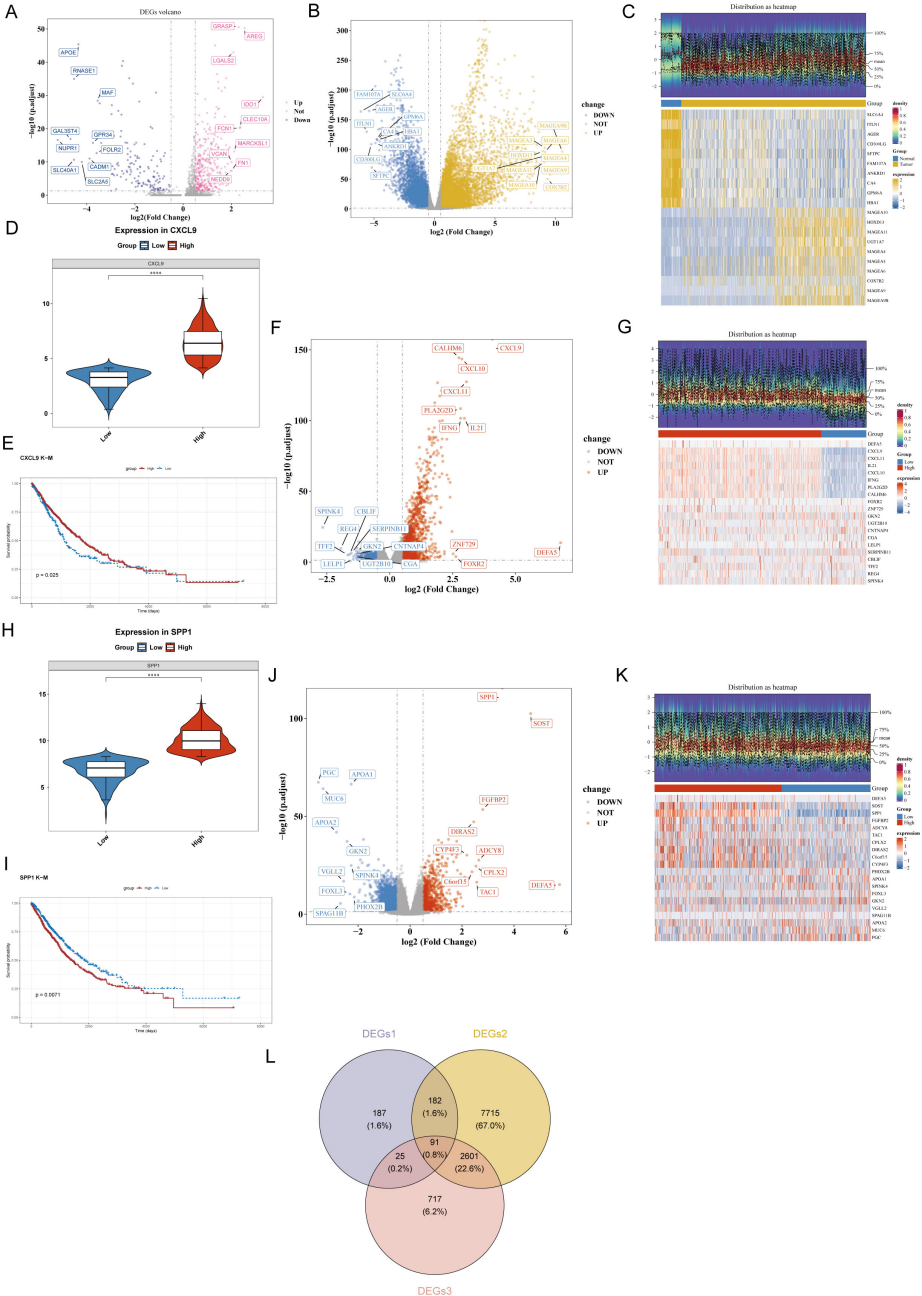
Acquisition of DEGs. **(A)** The distribution of 485 DEGs1 between CXCL9^+^SPP1^−^ and CXCL9^−^SPP1^+^ macrophages was analyzed. **(B, C)** A total of 10,589 DEGs2 in the TCGA training cohort were identified. **(D)** Differential expression of CXCL9 between the high and low expression groups was detected. **(E)** Significant differences were observed in the K-M survival curves between the CXCL9 high and low expression groups. **(F, G)** A total of 1,838 DEGs between the CXCL9 high and low expression groups were identified. **(H)** Differential expression of SPP1 between the high and low expression groups was detected. **(I)** Significant differences were found in the KM survival curves between the SPP1 high and low expression groups. **(J, K)** A total of 2,100 DEGs between the SPP1 high and low expression groups were identified. **(L)** A Venn diagram was constructed to show the overlap of 91 intersecting genes.

To screen representative DEGs in tumor-associated macrophages and identify key regulatory genes, we compared the gene expression profiles of *CXCL9*^+^*SPP1*^-^ Mac and *CXCL9*^-^SPP1^+^ Mac in tumor samples. A total of 485 significantly DEGs (DEGs1) were identified, of which 340 genes were upregulated and 145 genes were downregulated in the CXCL9^+^SPP1^-^ Mac population (**Figure 5A**).

### Transcriptomic differential analysis of NSCLC based on the TCGA cohort

To identify DEGs between tumor and control groups in the TCGA training set, volcano plot analysis revealed 10,589 significantly DEGs (DEGs2), including 6,907 upregulated and 3,683 downregulated genes in tumors (**Figure 5B**). The expression patterns of the top 10 most significantly upregulated and downregulated genes were presented in a heatmap (**Figure 5C**).

### Clinical significance and transcriptomic impact of *CXCL9* and *SPP1* expression levels

To evaluate differences in *CXCL9* expression between high- and low-expression groups, tumor samples in the TCGA training set were divided into high-expression (n = 789) and low-expression (n = 219) groups based on the optimal *CXCL9* expression cutoff value (4.146093). *CXCL9* expression differed significantly between the two groups (**Figure 5D**). Survival curves stratified by CXCL9 expression with significant differential expression, with the high-expression group exhibiting higher survival probability (**Figure 5E**). DEGs between CXCL9 high and low expression groups were analyzed using the DESeq2 package, resulting in 1,838 DEGs, including 431 downregulated and 1,407 upregulated genes in the high-expression group (**Figure 5F**). A heatmap was generated to display the top 10 upregulated and downregulated genes based on |log2FoldChange| (**Figure 5G**).

For *SPP1*, tumor samples in the TCGA training set were divided into high-expression (n = 593) and low-expression (n = 415) groups based on the optimal cutoff value (8.295627). *SPP1* expression differed significantly between the two groups (**Figure 5H**). Survival analysis showed that the low-expression group had significantly higher survival probability (**Figure 5I**). DEG analysis between *SPP1* high- and low-expression groups using DESeq2 identified 2,100 DEGs, with 1,324 downregulated and 776 upregulated genes in the high-expression group (**Figure 5J**). A heatmap displayed the top 10 changed genes (**Figure 5K**). The union of DEGs from *CXCL9* and *SPP1* groups was obtained after deduplication, resulting in 3,434 genes (DEGs3).

### Acquisition of intersection genes

To screen core genes with pivotal roles in NSCLC development from a multi-omics perspective, we integrated three key DEG sets obtained from previous analyses: macrophage polarization-related genes from single-cell data (DEGs1, 485 genes), tumor-normal tissue DEGs from TCGA data (DEGs2, 10,589 genes), and *CXCL9/SPP1* expression-related DEGs (DEGs3, 3,434 genes). Venn diagram analysis of the intersection of these three sets identified 91 common core genes (**Figure 5L**).

### GSEA of *CXCL9* and *SPP1* reveals their potential functional differences

**Figure 6:**
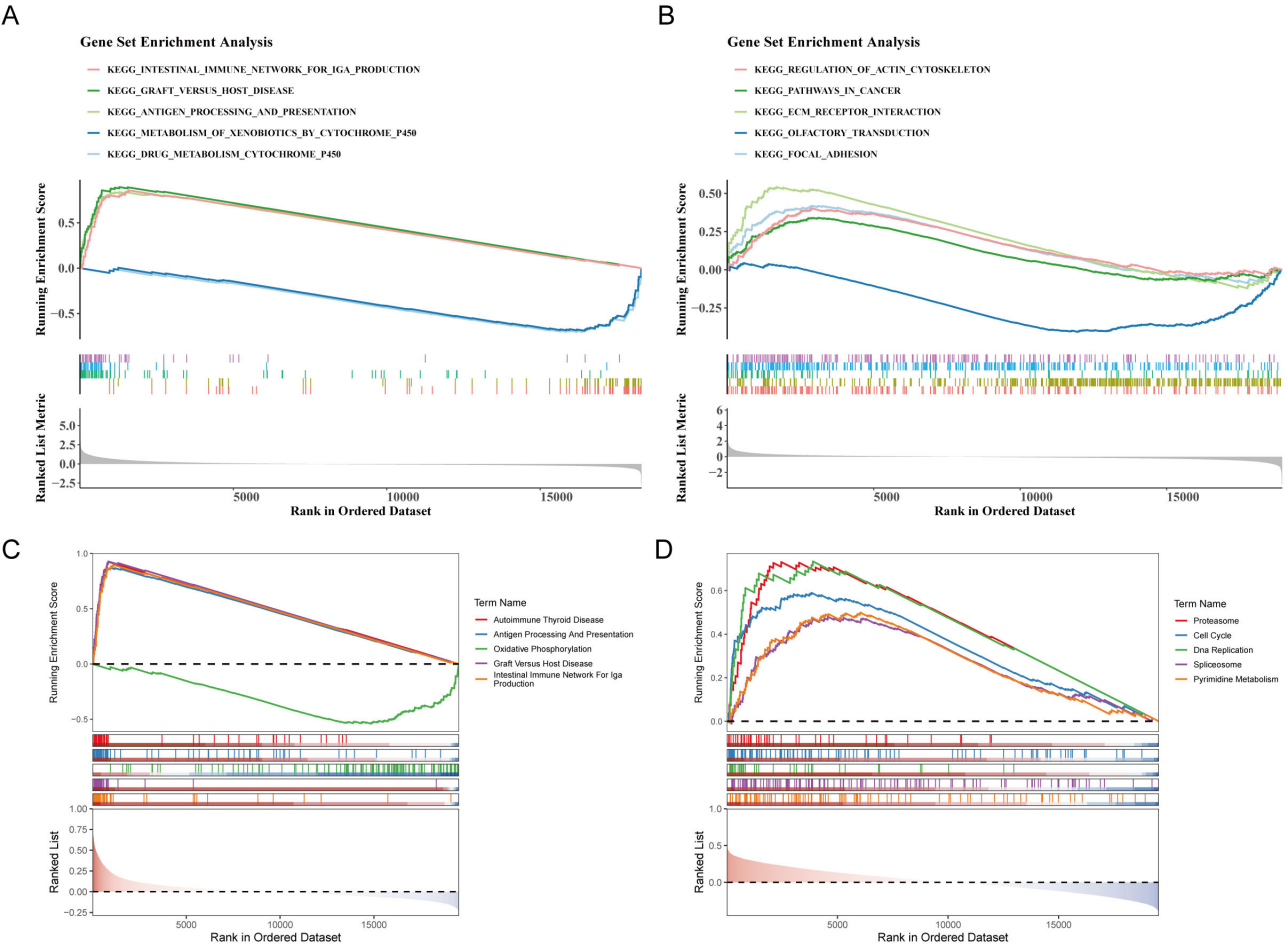
Gene set enrichment analysis (GSEA) of CXCL9 and SPP1 high/low expression groups. **(A)** GSEA was performed between the CXCL9 high- and low-expression groups, and 41 KEGG pathways were significantly enriched. **(B)** GSEA was conducted between the SPP1 high- and low-expression groups, and 14 KEGG pathways were significantly enriched. **(C, D)** Single−gene GSEA was performed for CXCL9 and SPP1, and 54 and 51 KEGG pathways were significantly enriched, respectively.

To systematically explore the biological pathways and functions of *CXCL9* and *SPP1* in NSCLC, GSEA was performed. Using the differential expression analysis between *CXCL9* high and low expression groups, 41 KEGG pathways were significantly enriched in the *CXCL9* high-expression group. The top five most significantly enriched pathways included Drug Metabolism - Cytochrome P450, Xenobiotic Metabolism - Cytochrome P450, Antigen Processing and Presentation, Graft-versus-Host Disease, and Intestinal Immune Network for IgA Production (**Figure 6A**). A Similar analysis for *SPP1* revealed that 14 pathways were significantly enriched in its high-expression group, with the top five including Focal Adhesion, Olfactory Transduction, ECM-Receptor Interaction, Pathways in Cancer, and Regulation of Actin Cytoskeleton (**Figure 6B**).

Additionally, single-gene GSEA analysis was conducted on all samples in the TCGA cohort to evaluate the above findings. CXCL9 correlation analysis enriched 54 pathways, with the most significant being Autoimmune Thyroid Disease, Antigen Processing and Presentation, Oxidative Phosphorylation, Graft-versus-Host Disease, and Intestinal Immune Network (**Figure 6C**). SPP1 correlation analysis enriched 52 pathways, with the top ones being Proteasome, Cell Cycle, DNA Replication, Spliceosome, and Pyrimidine Metabolism (**Figure 6D**).

### Screening of prognostic genes and model construction

**Figure 7:**
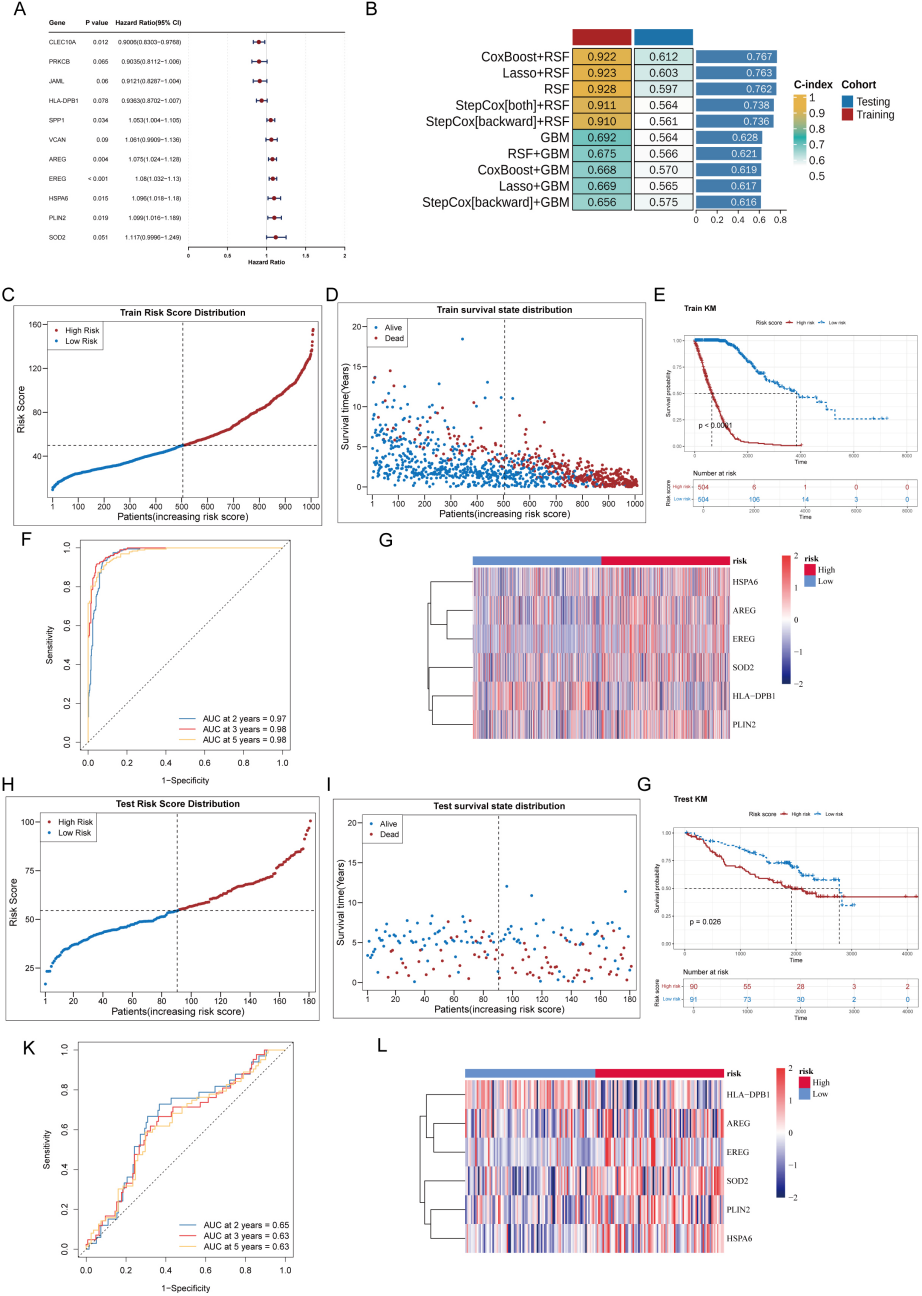
Screening of prognostic genes and construction/validation of a risk prognostic model. **(A)** A forest plot of univariate Cox proportional hazards regression analysis was generated, and 11 prognosis-related genes were identified. **(B)** The top 10 models ranked by C-index among 10 machine learning algorithm combination models were shown (the combined model of CoxBoost + RSF was adopted). **(C)** The distribution of risk scores in the TCGA training cohort was analyzed. **(D)** The distribution of survival outcomes in tumor samples between the high- and low-risk groups was displayed (TCGA training cohort; the proportion of deceased patients was markedly increased with elevated risk scores). **(E)** Significant differences were observed in the Kaplan−Meier (KM) survival curves between the high- and low-risk groups (TCGA training cohort). **(F)** ROC curves for 2−, 3− and 5−year overall survival were plotted (AUC values >0.6 in the TCGA training cohort). **(G)** A heatmap of the expression levels of prognostic genes between the high- and low-risk groups was constructed (TCGA training cohort; AREG, EREG, PLIN2, HSPA6 and SOD2 were upregulated in the high-risk group, whereas HLA−DPB1 was highly expressed in the low-risk group). **(H)** The distribution of risk scores in the GSE50081 validation cohort was analyzed. **(I)** The distribution of survival outcomes in tumor samples between the high- and low-risk groups was displayed (GSE50081 validation cohort; the proportion of deceased patients was markedly increased with elevated risk scores). **(J)** Significant differences were found in the KM survival curves between the high- and low-risk groups (GSE50081 validation cohort). **(K)** ROC curves for 2−, 3− and 5−year overall survival were plotted (AUC values >0.6 in the GSE50081 validation cohort). **(L)** A heatmap of the expression levels of prognostic genes between the high- and low-risk groups was constructed (GSE50081 validation cohort; AREG, EREG, PLIN2, HSPA6 and SOD2 remained highly expressed in the high-risk group, whereas HLA−DPB1 was significantly upregulated in the low-risk group).

To screen core genes with clinical prognostic value from the 91 common genes and construct a robust prediction model, systematic analysis was performed using the TCGA training set. Univariate Cox regression analysis initially identified 11 prognosis-related genes from the 91common genes (**Figure 7A**). PH assumption test confirmed seven genes that met the PH assumption (p > 0.05) as stable potential prognostic markers, including *AREG*, *EREG*, *HLA-DPB1*, *HSPA6*, *PLIN2*, *SOD2*, and *VCAN* (**Supplementary Table 3**). To establish the optimal prediction model, 101 algorithm combinations were constructed using ten machine learning methods, and predictive performance was evaluated in the TCGA training set and GSE50081 validation set using the leave-one-out cross-validation (LOOCV) framework. Based on C-index ranking, the CoxBoost+RSF combination, which showed the best performance in both the training and validation sets (average C-index > 0.6), was selected as the final model (**Figure 7B**). This model included 6 core prognostic genes: *AREG*, *EREG*, *HLA-DPB1*, *PLIN2*, *HSPA6*, and *SOD2*. These genes constituted a robust CS-polarity-associated TME signature, which was used for subsequent risk score construction and validation.

### Construction and validation of the prognostic risk model

Using the six screened prognostic genes, a prognostic risk model was constructed in the TCGA training set using the optimal CoxBoost+RSF algorithm. Using the median risk score (49.80) as the cutoff, patients were divided into high-risk (n = 504) and low-risk (n = 504) groups. Risk distribution and survival status plots showed that the proportion of deceased patients increased with rising risk scores (**Figures 7C, D**). Kaplan-Meier analysis confirmed that patients in the low-risk group had significantly better OS than those in the high-risk group (**Figure 7E**). Time-dependent ROC curve analysis showed that the model achieved good accuracy in predicting 2-, 3-, and 5-year survival rates, with AUC values consistently exceeding 0.6 (**Figure 7F**). Gene expression heatmap further revealed that *AREG*, *EREG*, *PLIN2*, *HSPA6*, and *SOD2* were upregulated in the high-risk group, whereas *HLA-DPB1* was highly expressed in the low-risk group, consistent with their risk directions in Cox regression (**Figure 7G**).

To evaluate the universality and robustness of the constructed prognostic risk model, comprehensive evaluation was performed using the independent GSE50081 dataset (n = 181). Using the same model parameters as the training set, risk scores were calculated, and patients were divided into high-risk (n = 90) and low-risk (n = 91) groups based on the median risk score (54.56). Risk distribution visualization showed that the proportion of deceased patients increased with rising risk scores, closely mirroring the pattern in the training set (**Figures 7H, I**). Survival analysis further confirmed the predictive efficacy of the model. Kaplan-Meier curves showed that patients in the low-risk group had significantly better OS than those in the high-risk group, which reproduced the core findings of the training set in the independent validation cohort (**Figure 7J**). Time-dependent ROC curve analysis indicated that the model maintained good accuracy in predicting 2-, 3-, and 5-year survival rates, with AUC values stably above 0.6, demonstrating its predictive ability across different time points (**Figure 7K**). Notably, the expression patterns of the six prognostic genes in the validation set were consistent with those in the training set: *AREG*, *EREG*, *PLIN2*, *HSPA6*, and *SOD2* were highly expressed in the high-risk group, whereas *HLA-DPB1* was upregulated in the low-risk group (**Figure 7L**).

### Correlation analysis between risk score and clinical features

**Figure 8:**
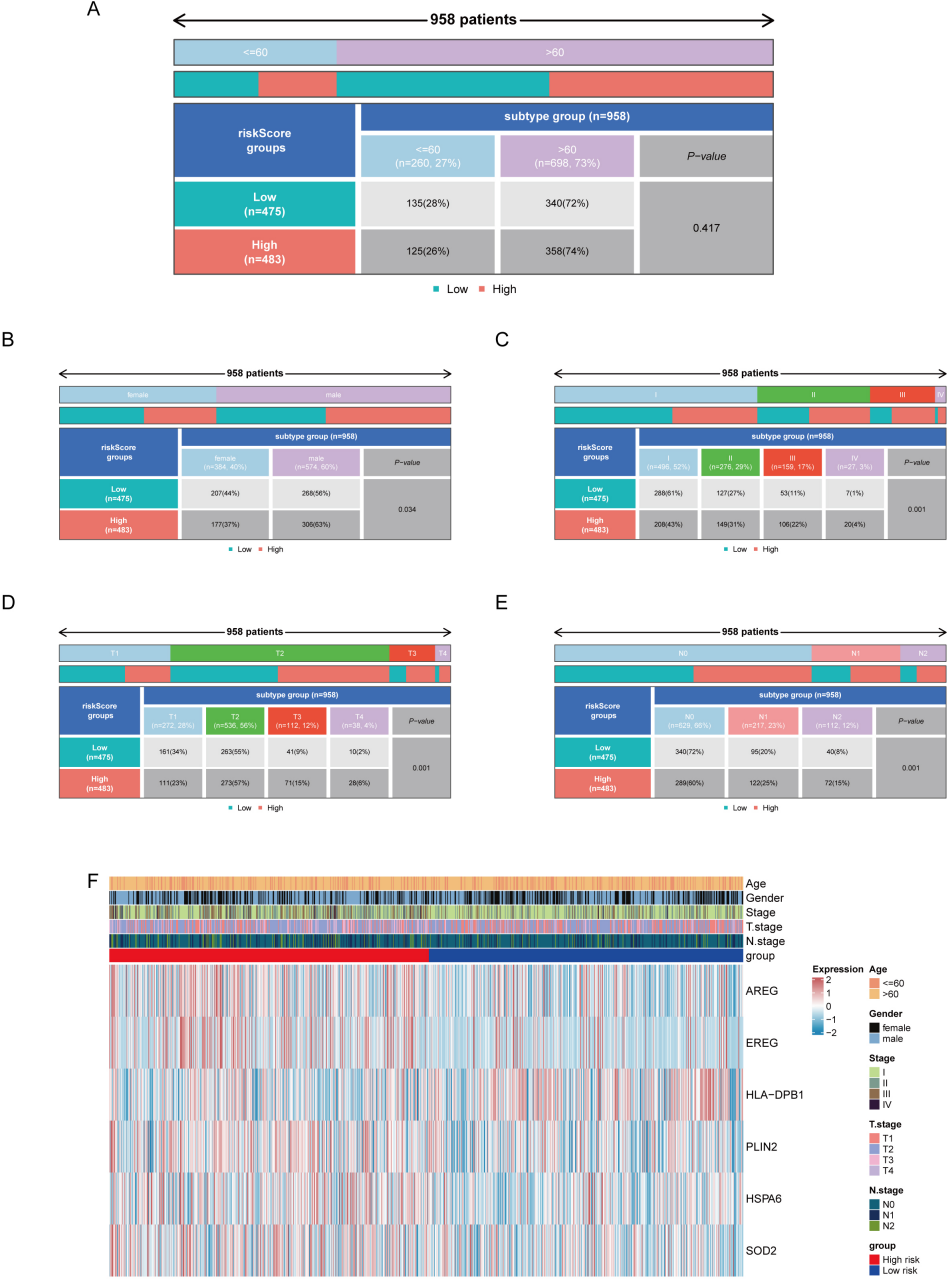
Correlation analysis between risk scores and clinical features. **(A–E)** Relationships between different clinical features and high/low risk groups. **(F)** Distribution of high and low risk patients across different clinical features.

To analyze the relationship between clinicopathological features and risk groups, systematic analysis was performed using data from 958 patients with complete clinical information in the TCGA training set. Chi-square test showed that risk stratification was significantly correlated with gender, T stage, N stage, and overall stage, but not with age (**Figures 8A–E**). Heatmap visualization further revealed the correlation pattern between risk score and clinical features: age groups were evenly distributed between high- and low-risk groups; female patients were more frequently in the low-risk group; patients with stage I–II were mostly in the low-risk group, whereas those with stage III–IV were predominantly in the high-risk group; in T stage, T1 patients were more in the low-risk group, T3 and T4 patients were more in the high-risk group, and T2 patients were evenly distributed; in N stage, N0 patients were more in the low-risk group, whereas N1 and N2 patients were more in the high-risk group; the gene *HLA-DPB1* was highly expressed in the low-risk group, whereas the other five prognostic genes (*AREG*, *EREG*, *PLIN2*, *HSPA6*, and *SOD2*) in the high-risk group (**Figure 8F**).

### Correlation analysis between risk model and tumor immune microenvironment

**Figure 9:**
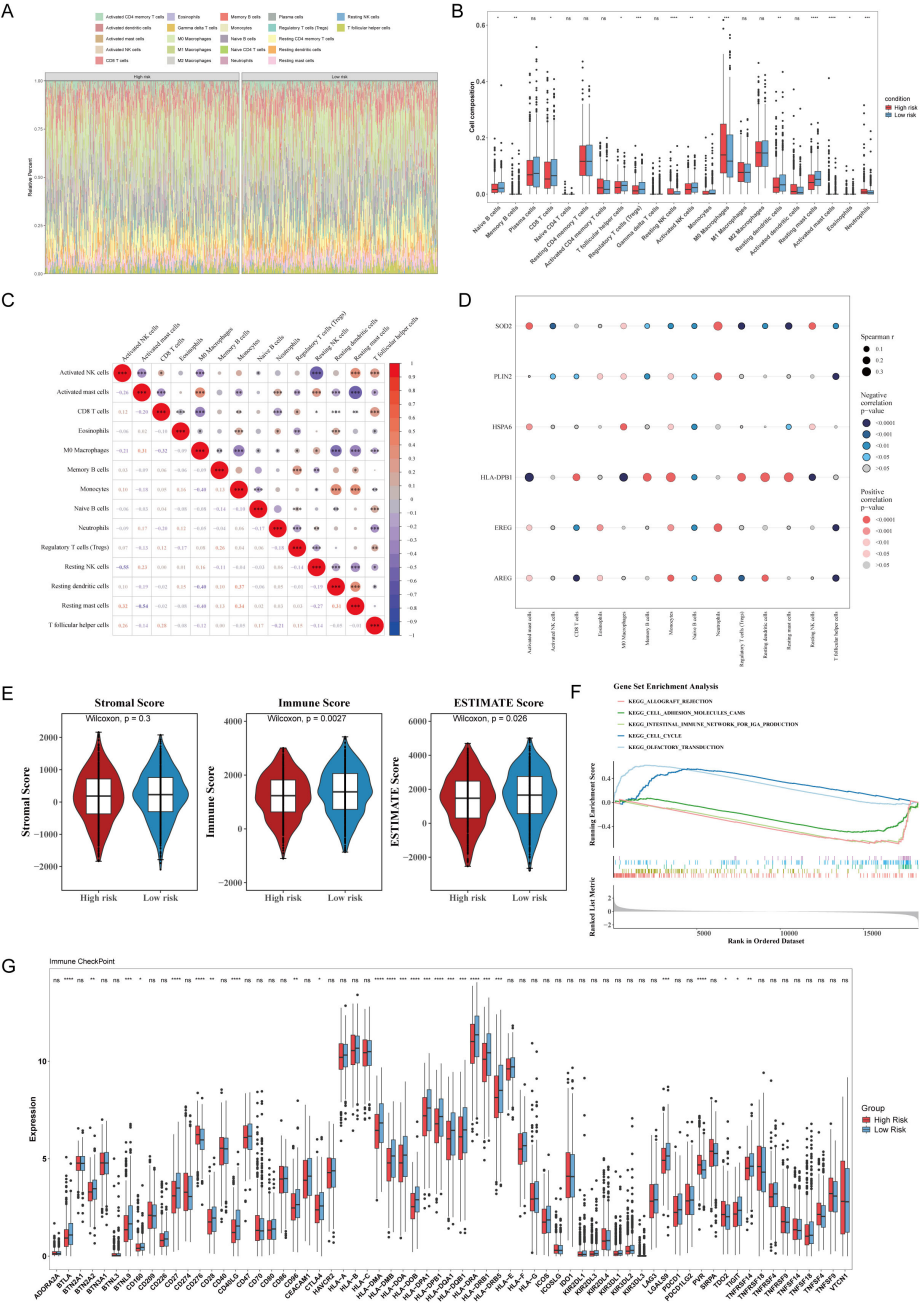
Immune infiltration analysis and GSEA. **(A)** The relative proportions of 22 immune cell types in the high− and low−risk groups were evaluated. **(B)** Fourteen differentially abundant immune cells between the high− and low−risk groups were identified. **(C)** Correlation analysis of the differentially abundant immune cells was performed (|r| > 0.3 and P < 0.05). **(D)** Correlation analysis between prognostic genes and differentially abundant immune cells was conducted (|r| > 0.3 and P < 0.05). **(E)** Differences in stromal scores, immune scores, and prognostic scores between the high- and low-risk groups were assessed. **(F)** GSEA results between the high- and low-risk groups were compared (TCGA training cohort). **(G)**The expression levels of immune checkpoints in the high− and low−expression groups were examined (P < 0.05).

To explore the association between the risk model and the tumor immune microenvironment, the CIBERSORT algorithm was used to analyze immune cell infiltration patterns in high- and low-risk groups. Immune cell composition analysis showed that M0 and M2 macrophages exhibited high infiltration levels in both groups, indicating the key role of macrophages in the TME (**Figure 9A**). Inter-group comparison revealed that the infiltration levels of 14 immune cell types differed significantly between high- and low-risk groups (**Figure 9B**).

Correlation analysis revealed a complex regulatory network among immune cells. Resting dendritic cells and monocytes showed the strongest positive correlation (cor = 0.37, p < 0.001), whereas resting NK cells and activated NK cells exhibited the strongest negative correlation (cor = –0.55, p < 0.001) (**Figure 9C**). Correlation analysis between prognostic genes and immune cells showed that HLA-DPB1 and monocytes had the strongest positive correlation (cor = 0.34, p < 0.001), whereas no strong negative correlation was observed (**Figure 9D**).

To explore the differences in tumor scores between high- and low-risk groups, visualization analysis revealed that the high-risk group had significantly higher immune scores and ESTIMATE scores compared with the low-risk group. However, no statistically significant difference was observed in stromal scores between the two groups (**Figure 9E**).

### Pathway enrichment characteristics of risk stratification

To dissect the potential biological function differences between the high- and low-risk groups, GSEA was performed using the TCGA training set. GSEA revealed significant differences in KEGG pathway enrichment between the two groups, identifying 14 significantly enriched pathways. The top five most significantly enriched pathways were KEGG_OLFACTORY_TRANSDUCTION, KEGG_CELL_CYCLE, KEGG_INTESTINAL_IMMUNE_NETWORK_FOR_IGA_PRODUCTION, KEGG_CELL_ADHESION_MOLECULES_CAMS, and KEGG_ALLOGRAFT_REJECTION (**Figure 9F**).

Differential Analysis of Immune Checkpoints: Analysis of immune checkpoint gene expression differences between the high- and low-risk groups in the TCGA training set identified 26 immune checkpoints with significant differential expression, including *BTLA*, *BTN2A2*, *BTNL9*, *CD160*, and *HLA-DPB1*. To identify candidate immune checkpoint genes with potential clinical value, we focused on those with high statistical significance. A total of 18 immune checkpoints, including *BTLA*, *BTNL9*, *CD27*, *CD276*, and *HLA-DPB1*, met the criterion of p < 0.001, providing a potential theoretical basis for optimizing immunotherapeutic strategies (**Figure 9G**).

**Figure 10:**
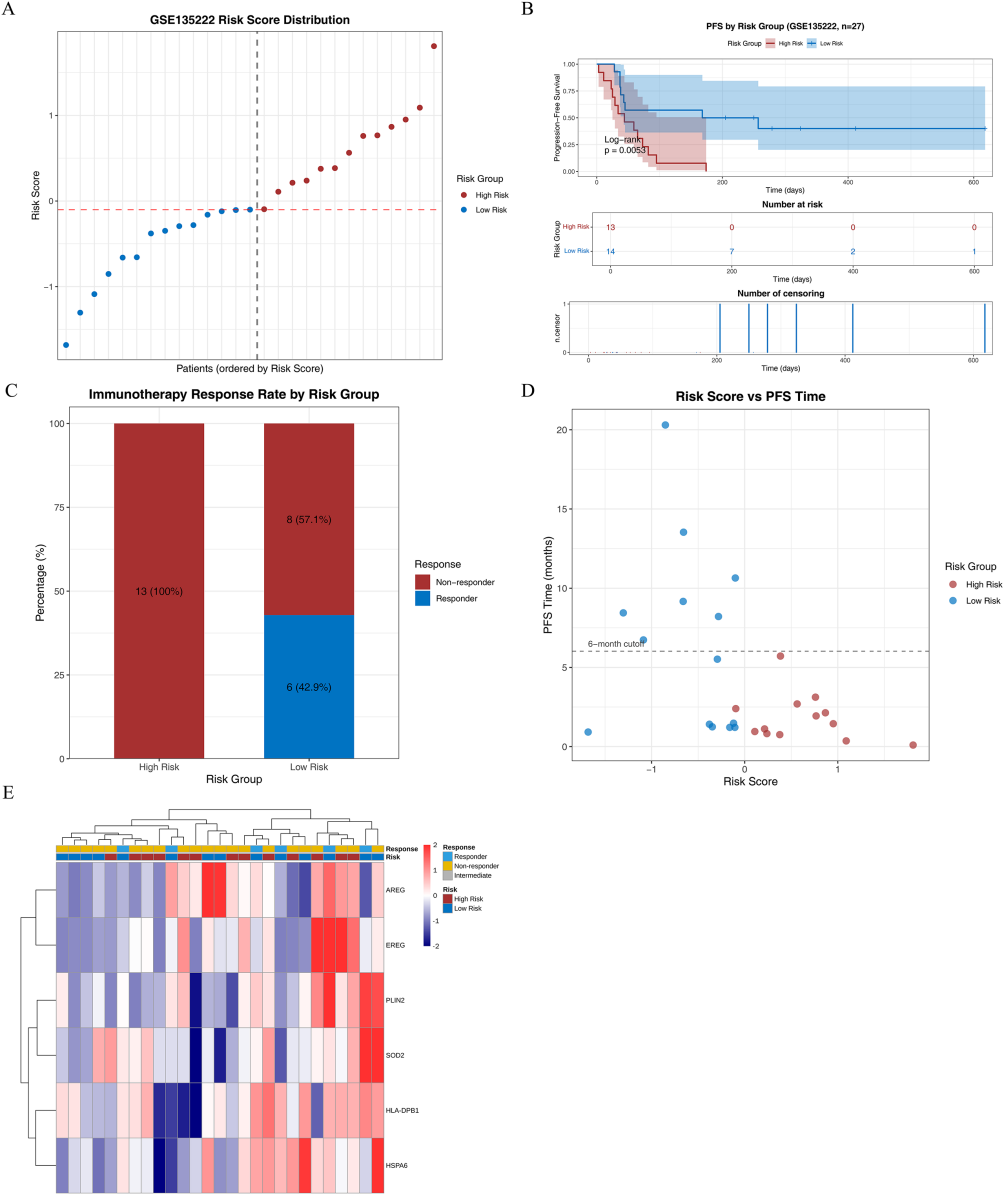
Immunotherapy response. **(A)** Distribution and risk stratification of Risk Score in GSE135222 dataset; **(B)** Survival analysis; **(C)** Bar plot of immunotherapy response rate; **(D)** Scatter plot of Risk Score versus PFS time; **(E)** Heatmap of signature gene expression.

In the GSE135222 cohort, samples were divided into high-risk (n = 13) and low-risk (n = 14) groups according to the median cutoff value of -0.1008.A significant survival difference was observed between the two groups (p = 0.0053).All patients in the high-risk group were classified as non−responders, whereas six responders were identified in the low−risk group. No response of EREG was detected in the high-risk group (**Figures 10A–E**).

### Somatic mutation analysis

**Figure 11:**
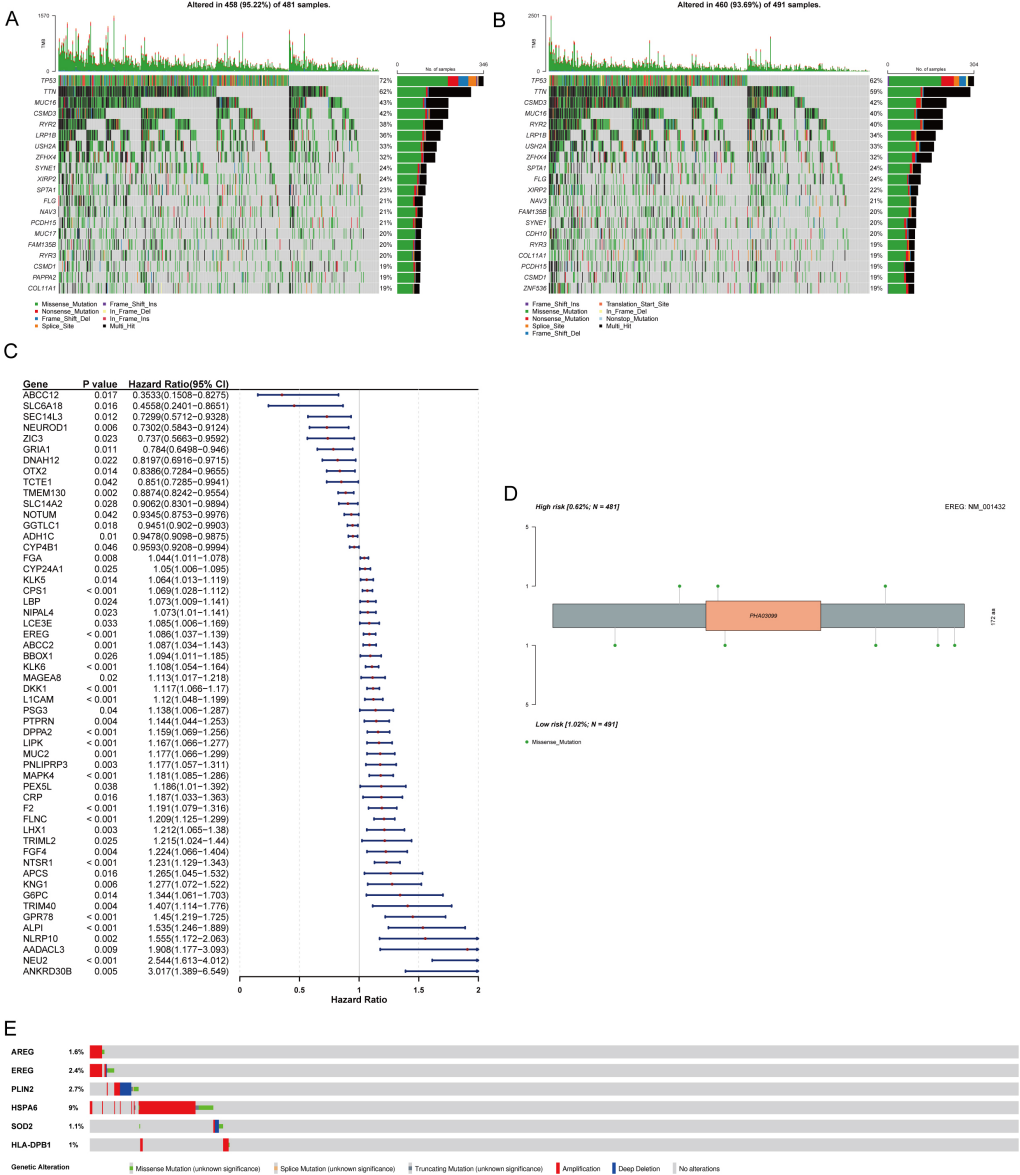
Tumor mutation analysis. **(A)** Somatic mutations in tumors of the high risk group (the mutation rate of TP53 was the highest). **(B)** Somatic mutations in tumors of the low risk group (the mutation rate of TP53 was the highest). **(C)** Forest plot of mutated genes from univariate Cox regression analysis. **(D)** Lollipop plot showing the distribution of EREG mutation sites in high and low risk samples. **(E)** Mutation status of prognostic genes.

To explore differences in genomic mutation characteristics between the high- and low-risk groups, waterfall plots were used to visualize the mutation profiles of the top 20 genes with the highest mutation frequency in each group. *TP53* was the most frequently mutated gene in both groups, with missense mutations being the predominant type (**Figures 11A, B**). To identify differentially mutated genes between the two groups, 10,662 genes with mutations in at least five samples were compared. Differential analysis using DESeq2 identified 125 differentially mutated genes, of which 62 were enriched in the high-risk group and 63 in the low-risk group.

Univariate Cox proportional hazards regression analysis of these differentially mutated genes identified 54 genes significantly associated with prognosis. Among these, 15 genes (e.g., *ABCC12*, *SLC6A18*) were protective factors (HR < 1), whereas 39 genes (e.g., *ANKRD30B*, *NEU2*) were risk factors (HR > 1) (**Figure 11C**). These 54 genes were subjected to PH assumption test, and 43 key genes meeting the PH assumption (p > 0.05) were identified (**Supplementary Table 4**). To analyze the biological impact of key mutated genes, the prognostic gene *EREG* was selected, and its mutation sites within protein domains were visualized using lollipop plots. Mutations in *EREG* were predominantly missense mutations (**Figure 11D**). To explore the mutation status of prognostic genes, mutation analysis was performed using NSCLC patient data from the cBioPortal database. Waterfall plots showed that *HSPA6* had the highest mutation rate, followed by *PLIN2* (**Figure 11E**).

### Pseudotime analysis predicts potential differentiation trajectories of macrophage subpopulations

Most prognostic genes were shown to be highly expressed in macrophages by bubble plots and violin plots (**Supplementary Figures 6A,B**). To dissect the potential differentiation trajectory of macrophage subpopulations and the expression dynamics of key genes, Monocle pseudotime analysis was performed on annotated macrophage subpopulations in the GSE198099 dataset. It should be noted that this represents a computationally inferred trajectory based on transcriptomic similarity, rather than real cell lineage tracing. The results suggested that macrophages after DDRTree dimensionality reduction potentially exhibited clear clustering and trajectory distribution in two-dimensional space (Component 1/Component 2). Based on the combined expression of *CXCL9* and *SPP1*, macrophages were classified into four subpopulations: *CXCL9*^+^*SPP1*^+^, *CXCL9*^+^*SPP1*^−^, *CXCL9*^−^*SPP1*^+^, and *CXCL9*^−^*SPP1*^−^. Each subpopulation exhibited regional aggregation in the trajectory plot, suggesting they correspond to different stages or functional states during differentiation.

**Figure 12:**
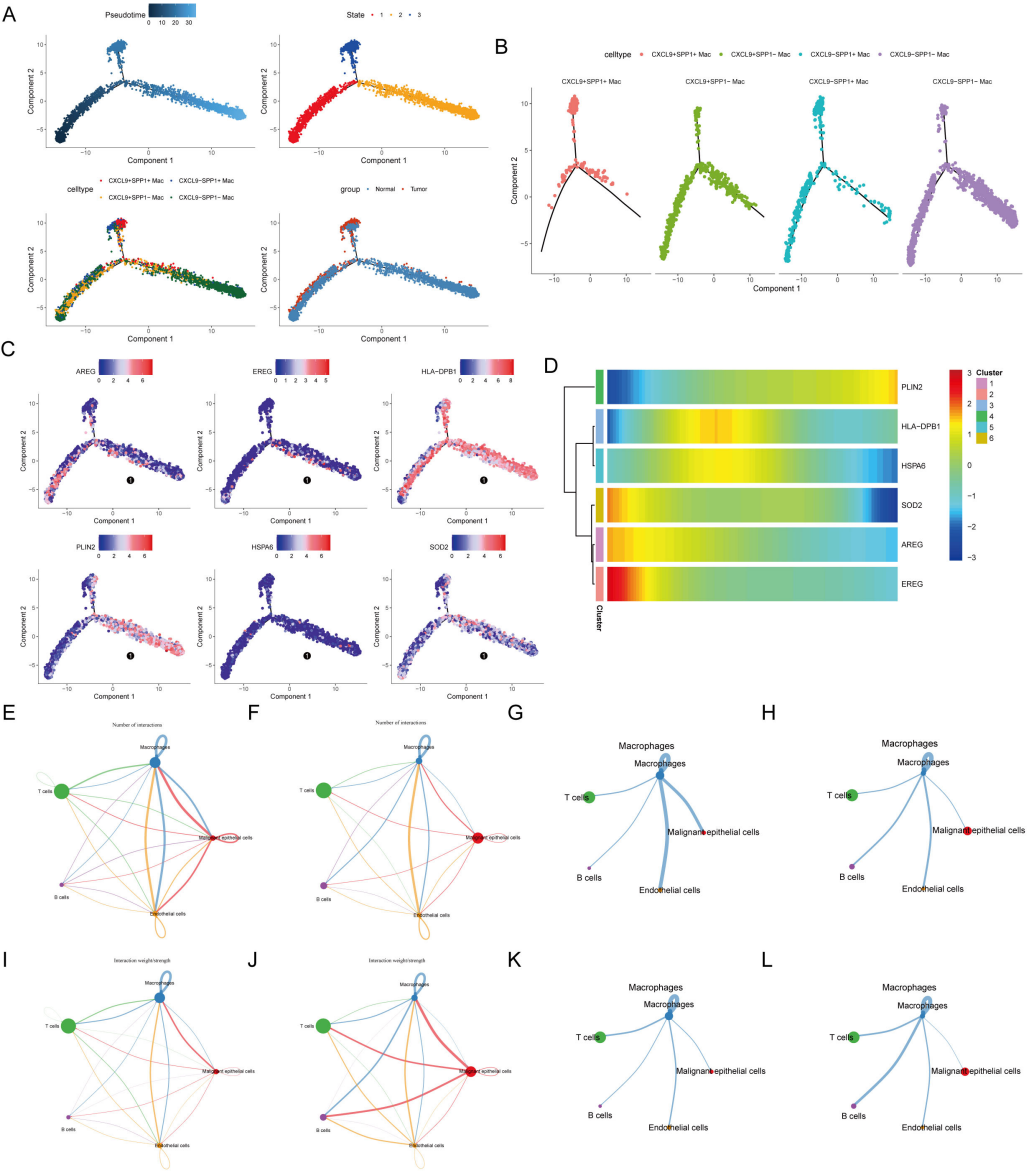
Pseudotime analysis and cell-cell communication of macrophages. **(A)** Pseudotime analysis of macrophages. **(B)** Analysis of pseudotime differentiation trajectories of macrophage subsets with different CXCL9-SPP1 phenotypes. **(C)** Spatial distribution characteristics of core functional gene expression in macrophage pseudotime trajectories. **(D)** Heatmap of expression profiles of key macrophage marker genes across different cell clusters. **(E)** Network diagram of the number of connections between different cell types in the control group. **(F)** Network diagram of the number of connections between different cell types in the tumor group. **(G)** Network diagram of the number of connections between macrophages and other cell types in the control group. **(H)** Network diagram of the number of connections between macrophages and other cell types in the tumor group. **(I)** Network diagram of connection weights between different cell types in the control group. **(J)** Network diagram of connection weights between different cell types in the tumor group. **(K)** Network diagram of connection weights between macrophages and other cell types in the control group. **(L)** Network diagram of connection weights between macrophages and other cell types in the tumor group.

Pseudotime analysis of macrophage subpopulations revealed that *CXCL9*^+^*SPP1*^+^ Mac were predominantly located in the middle stage of differentiation, *CXCL9*^-^*SPP1*^-^ Mac in the late stage, and *CXCL9*^+^*SPP1*^-^ Mac and *CXCL9*^-^*SPP1*^+^ Mac in the early-to-middle stage. Pseudotime analysis of tumor and control groups showed that tumor-derived macrophages were predominantly in the early-to-middle stage, whereas control-derived macrophages were distributed across all stages (**Figures 12A, B**). Analysis of prognostic gene expression levels during macrophage pseudotime revealed highly dynamic changes: *PLIN2* showed the highest expression level in the late developmental stage; *HLA-DPB1* and *HSPA6* showed low expression in the early stage, followed by an increase and then a gradual decrease in the late stage; *AREG*, *EREG*, and *SOD2* had high initial expression, which gradually decreased thereafter (**Figures 12C, D**).

### Intercellular communication network analysis infers potential ligand-receptor interactions

To computationally infer potential changes in intercellular interactions in the TME, the communication networks between all cell types in the control and tumor groups were analyzed based on the GSE198099 dataset. Importantly, these ligand-receptor interactions represent potential communication networks inferred from gene co-expression profiles, rather than physically validated protein binding.By comparing the total number of intercellular interactions, the number of connections from macrophages to B cells was higher in the tumor group, whereas connections between macrophages and endothelial cells, and between macrophages and malignant epithelial cells, were more frequent in the control group (**Figures 12E, F**). This trend was more pronounced in macrophage-specific communication diagrams: connections from macrophages to malignant epithelial cells and endothelial cells were more frequent in the control group, whereas connections from macrophages to B cells were most frequent in the tumor group (**Figures 12G, H**).

Analysis of interaction strength, measured by weight, revealed that the connection weight from malignant epithelial cells to macrophages was higher in the tumor group, whereas that from T cells to macrophages was higher in the control group (**Figures 12I, J**). From the macrophage perspective, signal output to B cells had the highest weight in the tumor group, whereas the signal output to malignant epithelial cells was stronger in the control group (**Figures 12K, L**).

**Figure 13:**
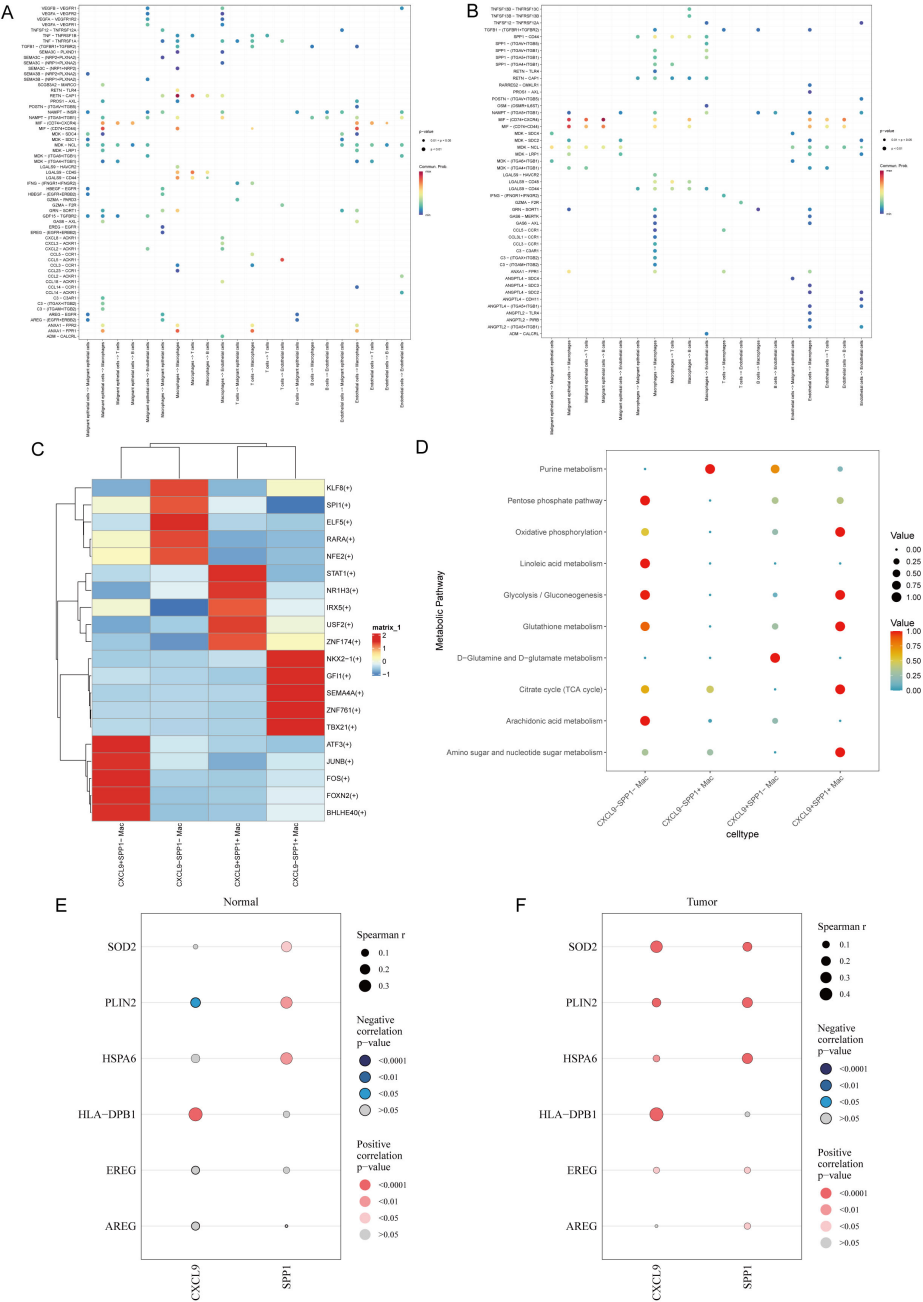
Ligand-receptor interactions and metabolic activity in cell-cell communication. **(A)** Bubble plot of ligand-receptor interactions in cell-cell communication across different cell types in the control group. **(B)** Bubble plot of ligand-receptor interactions in cell-cell communication across different cell types in the tumor group. **(C)** Normalized activity of the top five transcription factors (TFs) in macrophage subsets. **(D)** Bubble plot of metabolic activity in macrophage subsets. **(E, F)** Correlation between prognostic genes and CXCL9/SPP1.

Ligand-receptor pair bubble plots were used to dissect the communication basis between specific cell populations. The analysis inferred potentially more diverse ligand-receptor interactions in the control group. Among these, the PETN-CAP1 pair most likely mediated autocrine communication between macrophages in the control group. in the tumor group, MIF secreted by malignant epithelial cells, through binding to its receptors (CD74 and CXCR4), most likely constituted the dominant pathway for signal transmission to B cells (**Figures 13A, B**).

### Single-cell transcription factor analysis

To understand the TFs and their regulated programs in the sequenced macrophage subpopulations, the enrichment of TF-bound genes in each subpopulation was analyzed. The following transcription factors exhibited high activity in each macrophage subpopulation: *CXCL9*^+^*SPP1*^+^ Mac: STAT1, *NR1H3, USF2, ZNF174*, and *IRX5*. *CXCL9*^+^*SPP1*^−^ Mac: *FOS, ATF3, FOXN2, BHLHE40*, and *JUNB*. *CXCL9*^−^*SPP1*^+^ Mac: *SEMA4A, ZNF761, TBX21, NKX2-1*, and *GFI1*. *CXCL9*^−^*SPP1*^−^ Mac: *GFI1, ELF5, KLF8, RARA, NFE2*, and *SPI1* (**Figure 13C**).

### Transcriptomic prediction of metabolic pathway activity

To assess the metabolic status, functions, and potential regulatory mechanisms of key cells, metabolic activity analysis was performed. Of note these results represent transcriptomic predictions of metabolic pathway activity, rather than direct metabolite measurements. Transcriptomic scoring predicted the following metabolic activity patterns across macrophage subpopulations (**Figure 10D**): *CXCL9*^+^*SPP1*^+^ Mac: high activity in multiple energy metabolism and immune metabolism pathways, including the tricarboxylic acid (TCA) cycle and glutathione metabolism. *CXCL9*^+^*SPP1*^−^ Mac: high activity exclusively in D-glutamine and D-glutamate metabolism. *CXCL9*^−^*SPP1*^+^ Mac: high activity exclusively in purine metabolism. *CXCL9*^−^*SPP1*^−^ Mac: high activity in energy metabolism-related pathways, including glycolysis/gluconeogenesis and arachidonic acid metabolism (**Figure 13D**).

### Correlation analysis between *CXCL9/SPP1* and prognostic genes

Correlation analysis between prognostic genes and *CXCL9/SPP1* was performed. In the control group, *CXCL9* showed a significant positive correlation with *HLA-DPB1* (cor = 0.39, p < 0.05), whereas correlations with other prognostic genes were weak. *SPP1* showed weak correlations with all prognostic genes. In the tumor group, *CXCL9* had a significant positive correlation with *HLA-DPB1* (cor = 0.49, p < 0.05) and *SOD2* (cor = 0.36, p < 0.05), whereas correlations with other prognostic genes were weak (**Figures 13E, F**).

### Prognostic gene expression levels

**Figure 14:**
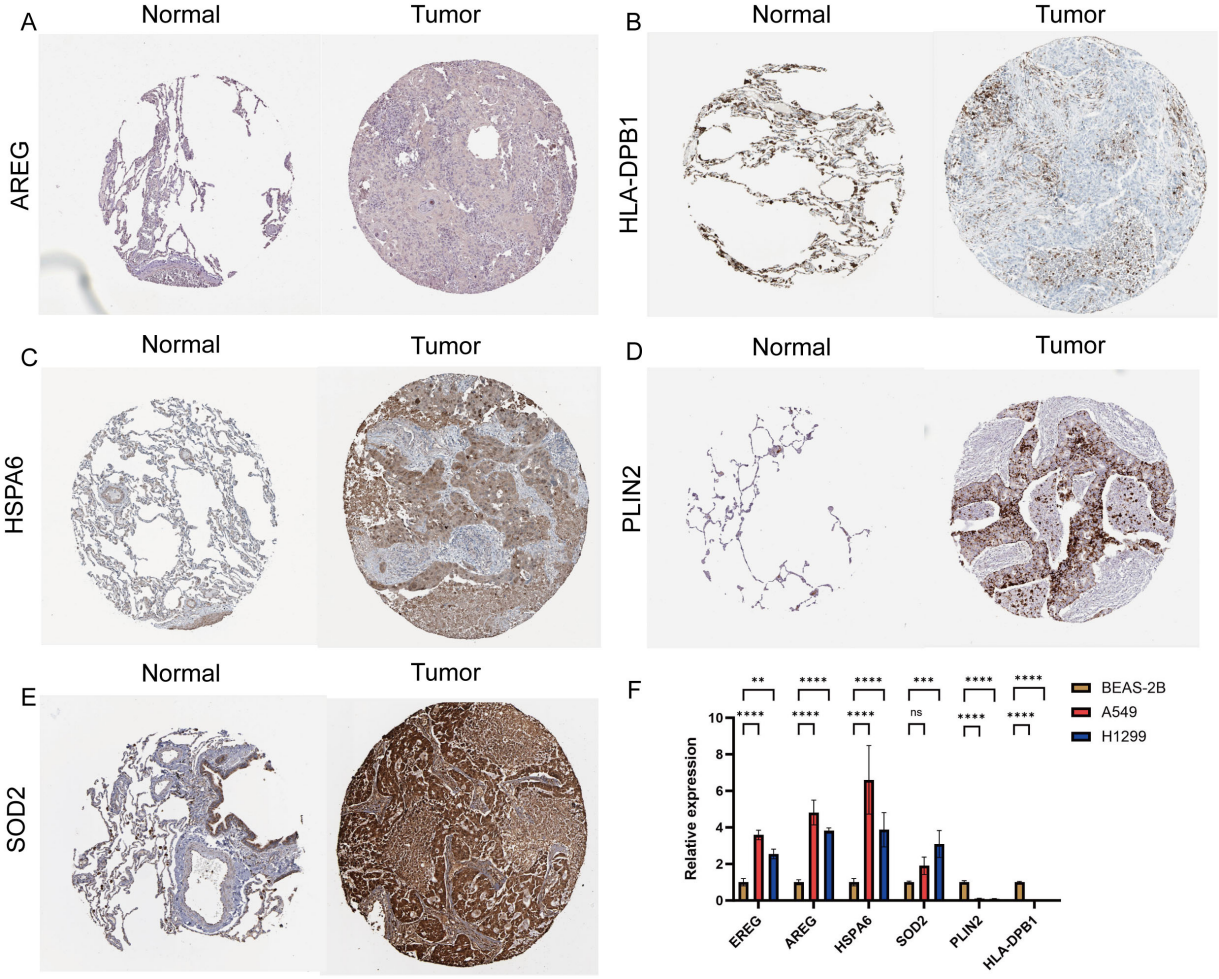
Prognostic gene expression levels. **(A–E)** Validation of prognostic genes by HPA protein. **(F)** Validation of prognostic gene RNA expression levels.

To evaluate prognostic gene expression at the protein level, the expression of target genes in NSCLC and normal tissues was analyzed using the HPA database. AREG was undetectable in normal tissues but showed moderate expression in tumor tissues; HLA-DPB1 showed high expression in controls but was undetectable in tumor tissues; HSPA6 and PLIN2 were undetectable in controls but highly expressed in tumor tissues; SOD2 showed low expression in controls and high expression in tumor tissues. EREG was not detected (**Figures 14A–E**). Validating prognostic gene RNA expression levels by PCR. The expression levels of EREG, AREG, and HSPA6 in A549 and H1299 cells were all higher than those in BEAS-2B cells. The expression level of SOD2 in H1299 cells showed no significant difference compared with BEAS-2B cells. The expression levels of PLIN2 and HLA-DPB1 in A549 and H1299 cells were both lower than those in BEAS-2B cells (**Figure 14F**).

## Discussion

NSCLC is the most common histological subtype of lung cancer. Its marked heterogeneity and complex TME are key factors contributing to therapeutic resistance and poor prognosis (5). Among the diverse cellular components within the TME, TAMs have attracted considerable attention due to their high plasticity and pivotal role in immune regulation (22, 23). Traditionally, TAMs are categorized into anti-tumor M1 and pro-tumor M2 phenotypes. However, accumulating evidence indicates that this binary classification fails to capture the continuous functional spectrum of TAMs in human tumors (24, 25). A landmark study by Bill et al. published in Science, which systematically analyzed head and neck squamous cell carcinoma (HNSCC), revealed that TAM polarity—defined by the *CXCL9/SPP1* (CS) expression ratio—serves as a core indicator that transcends traditional M1/M2 classification. This CS polarity is closely associated with patient prognosis and the overall immune status of the TME (25). Inspired by this finding, our study introduces the CS polarity axis into NSCLC research. Leveraging bulk transcriptomic and scRNA-seq data, we aimed to comprehensively investigate the prognostic value, immunoregulatory significance, and underlying molecular networks of CS polarity in NSCLC.

Although recent studies have established the CS polarity axis as a critical metric for evaluating tumor-associated macrophages (TAMs) across various cancers, its specific role and prognostic value in non-small cell lung cancer (NSCLC) have remained largely undefined. To bridge this gap and distinguish our work from existing TAM frameworks, this study introduces innovations across three dimensions. Regarding the research context, we elucidate the CS polarity axis within the unique tumor microenvironment of NSCLC. Methodologically, we mapped the dynamic plasticity of TAM polarization using single-cell pseudotime trajectory analysis and leveraged a combination of 101 machine learning algorithms to construct a robust CS-associated prognostic model. In terms of analytical depth, we extended beyond standard transcriptomic profiling to construct a multi-dimensional regulatory network integrating immune infiltration characteristics, metabolic pathway predictions, and cell-cell communication dynamics. Together, these approaches provide a specific, multidimensional, and applicable framework for evaluating TAM heterogeneity and patient outcomes in NSCLC.

Our study first confirmed the co-expression and mutual exclusivity of CXCL9 and SPP1 in NSCLC TAMs using scRNA-seq data, identifying four macrophage subpopulations with distinct CS expression patterns: *CXCL9*^+^*SPP1*^+^ macrophages (the double-positive subset with high expression of both the chemokine *CXCL9* and the tumor microenvironment-associated molecule SPP1),*CXCL9*^−^*SPP1*^−^macrophages (the double-negative subset with low expression of both markers, representing a relatively “resting” or inactive state), *CXCL9*^+^*SPP1*^−^macrophages (potentially exhibiting anti-tumor characteristics), and *CXCL9*^−^*SPP1*^+^macrophages (potentially exhibiting pro-tumor characteristics). This finding aligns with observations by Bill et al., suggesting that CS polarity is a universal characteristic of TAMs across different cancer types (16). Notably, we also detected a *CXCL9*^+^*SPP1*^+^ macrophage subpopulation in normal lung tissue, although its proportion and activation state differed significantly from those in tumor tissue. This implies that tumor-specific microenvironmental cues—such as local cytokine networks and hypoxia—are critical drivers of TAM polarization toward specific functional states (26). Differential expression analysis of the most representative *CXCL9*^+^*SPP1*^−^ and *CXCL9*^−^*SPP1*^+^ subpopulations in tumor tissue identified 485 genes (DEGs1) closely associated with CS polarity, laying the foundation for subsequent exploration of the core regulatory network.

By intersecting three gene sets, we identified 91 core genes consistently associated with NSCLC and CS polarity across multiple dimensions. This multi-step screening strategy narrowed the candidate gene pool, increasing the likelihood of identifying key drivers. Survival analysis confirmed the opposing prognostic values of *CXCL9* and *SPP1* in NSCLC: high *CXCL9* expression predicted favorable prognosis, whereas high *SPP1* expression correlated with poor outcomes—consistent with their known roles in immune regulation. CXCL9, an IFN-γ-induced chemokine, recruits effector lymphocytes such as CD8^+^ T cells and Th1 cells, shaping an immunologically “hot” TME (27, 28). In contrast, SPP1 (osteopontin) promotes tumor progression through multiple mechanisms, M2-like macrophage polarization, tumor cell invasion and metastasis, and inducing immunosuppression (29, 30).

From a functional perspective, the biological roles of these genes closely align with the divergent directions of the CS polarity axis. HLA-DPB1, a key component of MHC class II molecules, plays a central role in antigen presentation and CD4^+^ T cell activation (31). Its high expression in the low-risk group is highly consistent with the adaptive immune activation driven by *CXCL9*^+^ anti-tumor TAMs, collectively shaping a “hot” tumor microenvironment conducive to cytotoxic T cell infiltration. This association is corroborated by our study: *HLA-DPB1* expression was significantly positively correlated with *CXCL9* and with the infiltration levels of multiple adaptive immune cell types.

In contrast, the five genes upregulated in the high-risk group reflect, from different dimensions, the microenvironmental characteristics shaped by SPP1^+^ pro-tumor TAMs. AREG and EREG, as EGFR ligands, are predominantly expressed in malignant epithelial cells. Given the established role of SPP1^+^ TAMs in activating downstream oncogenic signals through integrin and CD44 pathways, we speculate that SPP1^+^ TAMs may induce the upregulation of EGFR ligand expression in tumor cells via paracrine factors (e.g., TGF-β or IL-6), thereby establishing a pro-proliferative positive feedback loop between macrophages and tumor cells (32). This inference aligns well with previous reports on the role of AREG/EREG in driving malignant progression in KRAS-mutant NSCLC (33–35). HSPA6 and SOD2, as a heat shock protein and an antioxidant enzyme respectively, their high expression reflects an adaptive survival response of tumor cells to hypoxia and oxidative stress—typical features of the microenvironment shaped by SPP1^+^ TAMs (36–38). PLIN2, a lipid droplet-associated protein, its high expression suggests the activation of lipid metabolic reprogramming. This is consistent with the metabolic characteristics predicted for SPP1^+^ TAM-dominated microenvironments, reflecting an active state of fatty acid metabolism pathways that provide energy and synthetic precursors for tumor growth (39).

Patients were stratified into high- and low-risk groups based on the risk score derived from this CS-polarity-associated TME signature. Kaplan-Meier survival analysis clearly demonstrated that high-risk patients had significantly shorter OS. Further immune infiltration analysis revealed significant differences in TME immune cell composition between the two groups, with 14 immune cell types showing differential infiltration levels. The high-risk group exhibited features of an immunosuppressive microenvironment, whereas the low-risk group displayed stronger immune activity. This echoes the conclusions of Bill et al.’s study, where CS^+^ TAMs (high *CXCL9* expression) correlated positively with T cell, B cell, and dendritic cell infiltration, whereas CS^−^ TAMs (high *SPP1* expression) associated with immunosuppressive and pro-tumor pathways (16). Our results provide a transcriptomic rationale linking CS polarity-related molecular features to the overall immune status of the NSCLC TME.

Somatic mutation analysis further revealed that the mutation status of the prognostic gene EREG differed between high- and low-risk groups, and its mutation was associated with poorer prognosis (HR > 1). Mutations in the EREG gene may lead to a gain-of-function effect, resulting in the continuous activation of downstream oncogenic signaling pathways, such as PI3K/AKT and MEK/ERK (40, 41). This suggests that EREG may not only serve as a prognostic marker but that its mutations may also have functional significance. At the single-cell level, our intercellular communication analysis based on transcriptomic co-expression profiles inferred potentially enhanced connections between macrophages and other immune cells (e.g., B cells) in NSCLC tissue—suggesting that more active roles could be played by TAMs in regulating adaptive immune responses. Pseudotime analysis computationally modeled a potential differentiation trajectory of macrophage subpopulations with different CS polarity states: *CXCL9*^+^*SPP1*^−^ and *CXCL9*^−^*SPP1*^+^ subpopulations were primarily located in the early-to-middle stages of differentiation, CXCL9^+^SPP1^+^ subpopulations in the middle stage, and *CXCL9*^−^*SPP1*^−^ subpopulations tended toward the late stage. It was indicated that plasticity was exhibited by TAM polarity and dynamic alterations could be induced in response to TME signals. Pseudotime trajectory analysis revealed that TAM subpopulations in hepatocellular carcinoma follow two divergent differentiation trajectories: one toward *CXCL9*^+^ TAMs, which are associated with a favorable prognosis and likely exert anti-tumor effects by mediating T cell recruitment via the CXCL signaling pathway, and the other toward *SPP1*^+^ TAMs, forming an opposing axis in terms of their evolution (17).

Beyond the clinical prognostic model, this study uniquely delineates the highly NSCLC-specific transcriptional and metabolic regulatory networks underlying the CS polarity axis, distinctly differentiating our findings from broad pan-cancer models. whereas previous landmark studies have established the classical IFN-γ/STAT1 signaling as the pan-cancer driver of anti-tumor *CXCL9*^+^ TAMs, our single-cell inference reveals that within the unique NSCLC microenvironment, putative anti-tumor *CXCL9*^+^*SPP1*^−^ TAMs are not solely dependent on STAT1 (16). Instead, they are co-driven by STAT1 and stress-responsive factors like FOS, reflecting an adaptive transcriptional rewiring to the lung tumor microenvironment.

Metabolically, distinguishing from the classical pan-cancer “glycolysis-OXPHOS” TAM shift, our predictions uncover striking NSCLC specificities: the pro-tumorigenic *SPP1*^+^ subpopulation exhibits a specific hyperactivation in purine metabolism (42). This perfectly aligns with recent immunometabolic breakthroughs identifying aberrant purine metabolism (particularly immunosuppressive adenosine accumulation) as the core engine driving macrophage immune evasion and T cell exhaustion in lung cancer (43). Concurrently, the double-positive transitional state (*CXCL9*^+^*SPP1*^+^ TAMs) uniquely relies on glutathione metabolism, indicating a strict dependence on active redox homeostasis during polarization plasticity. Taken together, uncovering these NSCLC-specific transcriptional and metabolic vulnerabilities not only provides a high-resolution, organ-specific complement to pan-cancer TAM models but also generates highly translatable mechanistic hypotheses for targeted interventions, such as exploiting the purine metabolic axis in *SPP1*^+^ TAMs.

Despite the comprehensive analyses and promising findings, several limitations of this study merit attention. First, regarding study design, our reliance on retrospective public datasets introduces potential selection biases. Furthermore, the unstratified analysis of NSCLC may mask subtype-specific features of the CS polarity axis, highlighting the need for prospective, subtype-focused cohorts to validate these signatures. Second, regarding expression validation, although we provided preliminary experimental support via qRT-PCR and utilized the HPA database for protein-level insights, this study primarily relies on transcriptomics. The precise protein expression, post-translational modifications, and spatial distribution of key genes lack large-scale *in situ* clinical validation (e.g., multiplex immunohistochemistry). Third, and most importantly, it is crucial to emphasize that our mechanistic findings concerning differentiation trajectories, metabolic reprogramming, and intercellular communication are entirely derived from bioinformatic predictions. Therefore, these results should be strictly considered hypothesis-generating. Specifically, the cell-cell communication networks inferred from ligand-receptor co-expression have yet to be confirmed by physical interaction assays. Similarly, the causality of the predicted metabolic rewiring lacks direct evidence from *in vitro* gain/loss-of-function experiments and metabolic flux assays. Finally, the inferred immune crosstalk between CS-polarized TAMs and other immune cells (e.g., CD8^+^ T cells) necessitates future robust *in vivo* functional studies, such as macrophage depletion or TAM-tumor co-culture models, to definitively establish their mechanistic pathways and clinical translational value.

This study identifies the CS polarity axis of TAMs in NSCLC, including four functional subpopulations (e.g., macrophages with anti-tumor features *CXCL9*^+^*SPP1*^−^ and macrophages with pro-tumor features *CXCL9*^−^*SPP1*^+^). The CS-polarity-associated TME six-gene signature shows favorable prognostic stratification value, correlating with TME immune status, metabolic characteristics, and somatic mutations, thus providing potential directions for NSCLC prognostic assessment and immunotherapy focused on TAM polarity.

## Data Availability

The original contributions presented in the study are included in the article/**Supplementary Material**. Further inquiries can be directed to the corresponding authors.

## Supplementary material

Supplementary Figure 1 Single-cell data analysis. **(A, B)** Violin plots of nFeature RNA, nCount RNA, and percent.mt before and after quality control. **(C)** Screening of highly variable genes. **(D)** Results of principal component analysis for the single-cell dataset. **(E)** P-values of principal components calculated by the JackStraw function with permutation test. **(F)** Identification of usable dimensions.

Supplementary Figure 2 Visualization of quality control and normalization analysis for single-case spatial transcriptomic samples. **(A)** Paratumor sample of P11; **(B)** Tumor sample of P11; **(C**) Paratumor sample of P15; **(D)** Tumor sample of P15; **(E)** Paratumor sample of P19; **(F)** Tumor sample of P19; **(G)** Paratumor sample of P24; **(H)** Tumor sample of P24; **(I)** Paratumor sample of P25; **(J)** Tumor sample of P25.

Supplementary Figure 3 Quality control of spatial transcriptomics. **(A)** Elbow plot; **(B)** Spatial clustering plots of each paired sample; **(C)** UMAP dimensionality reduction and clustering plot of all integrated samples; **(D)** UMAP dimensionality reduction plot colored by sample group (Disease/Healthy).

Supplementary Figure 4 Marker gene selection. **(A)** Expression plots of marker genes; **(B)** Visualization of spatial expression distribution of marker genes in paired spatial transcriptomic samples.

Supplementary Figure 5 Spatial distribution characteristics of macrophage subtypes in paratumor and tumor tissues of NSCLC patients. **(A)** Paratumor sample of P11; **(B)** Tumor sample of P11; **(C)** Paratumor sample of P15; **(D)** Tumor sample of P15; **(E)** Paratumor sample of P19**;(F)** Tumor sample of P19; **(G)** Paratumor sample of P25; **(H)** Tumor sample of P25.

Supplementary Figure 6 Expression of prognostic genes in all cell types. **(A)** Bubble plot; **(B)** Violin plot.
