# Supplementary figures and images for: The CXCL9/SPP1 polarity axis in tumor-associated macrophages: immunoregulatory and prognostic significance in non-small cell lung cancer
Source: Front Immunol. 2026 Apr 17;17:1763652. doi: 10.3389/fimmu.2026.1763652 (PMC13132838; doi:10.3389/fimmu.2026.1763652)

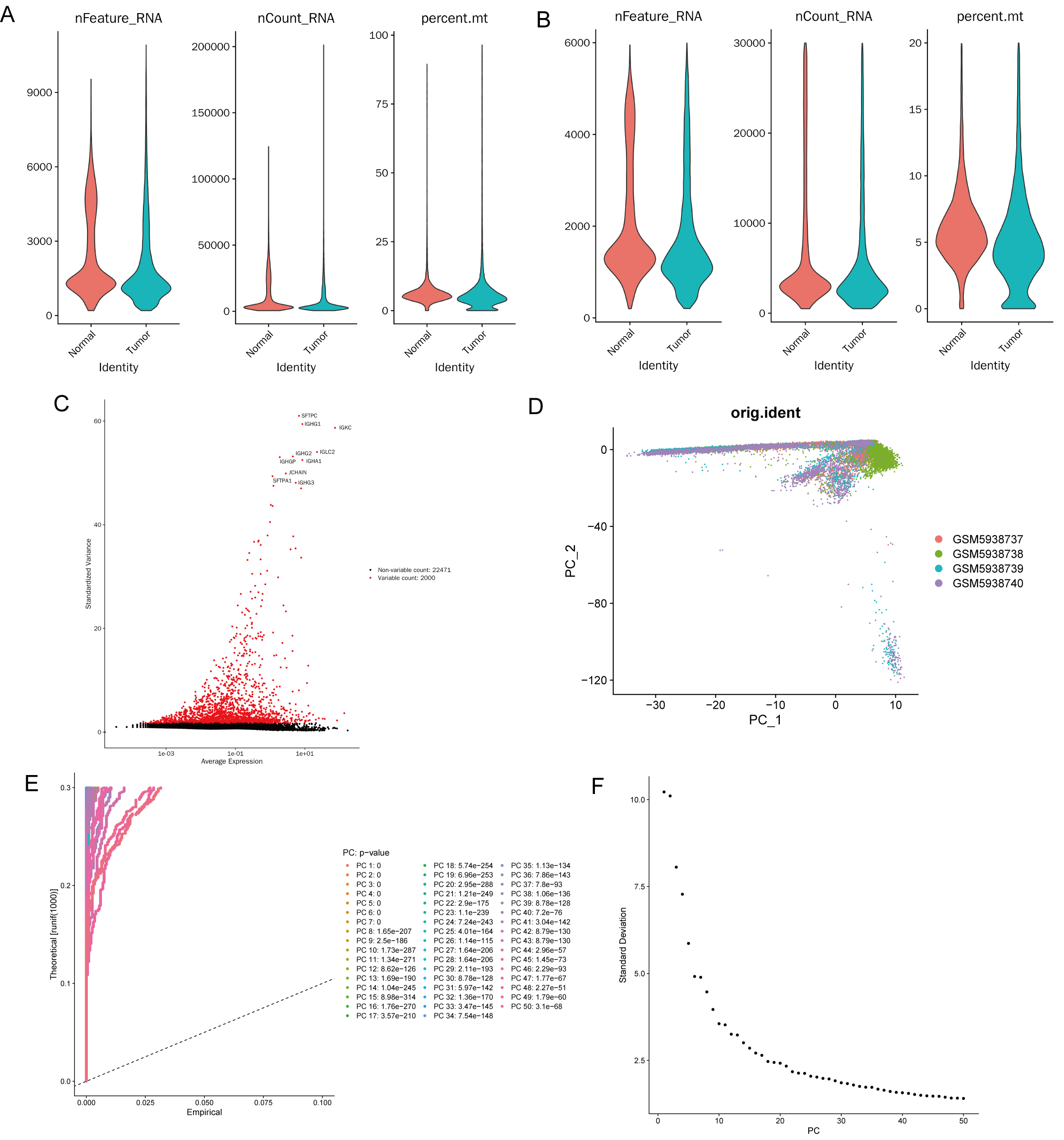

Supplement: Supplementary Figure 1 — Single-cell data analysis. (A, B) Violin plots of nFeature RNA, nCount RNA, and percent.mt before and after quality control. (C) Screening of highly variable genes. (D) Results of principal component analysis for the single-cell dataset. (E) P-values of principal components calculated by the JackStraw function with permutation test. (F) Identification of usable dimensions. [file Image1.jpeg]

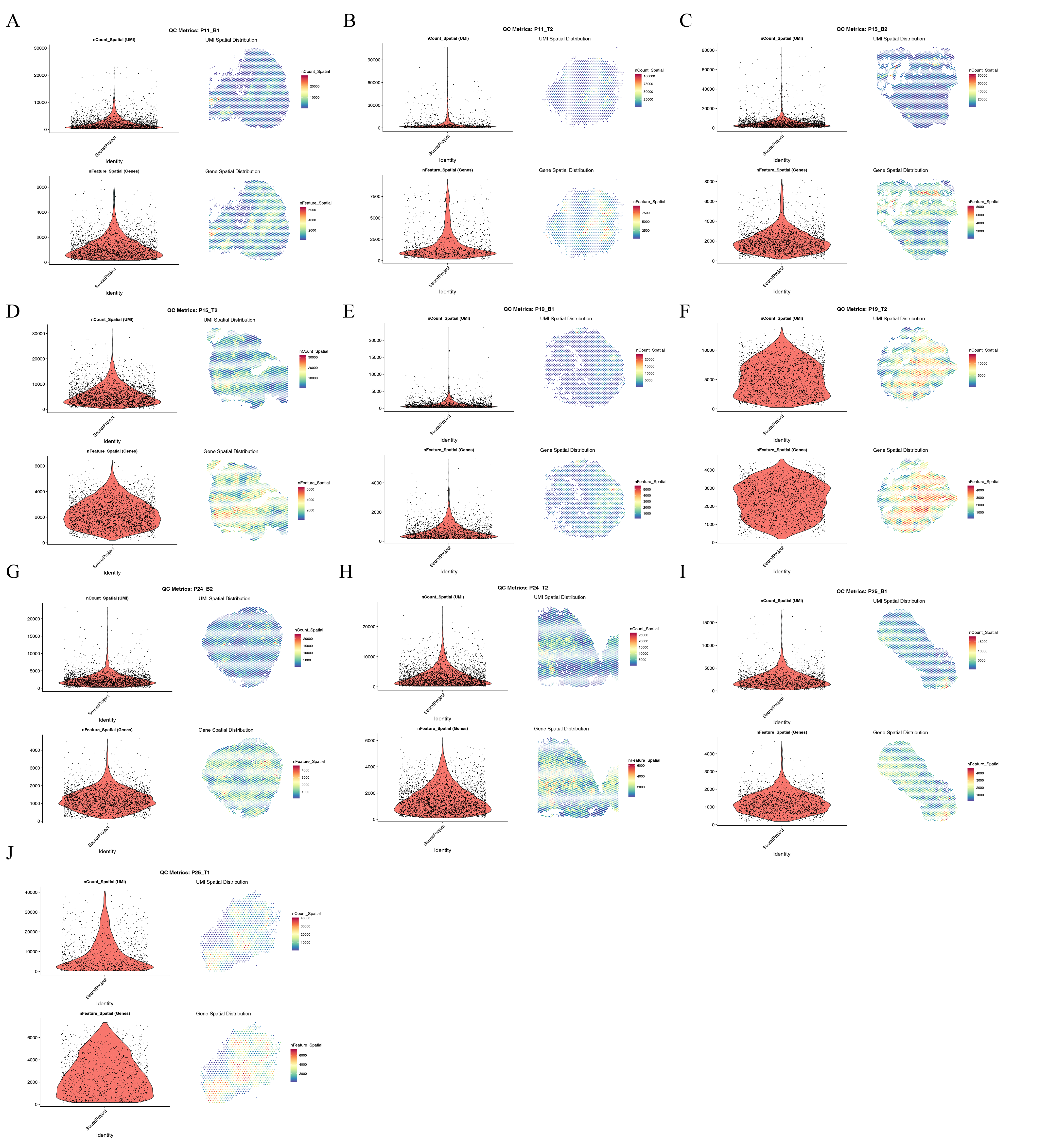

Supplement: Supplementary Figure 2 — Visualization of quality control and normalization analysis for single-case spatial transcriptomic samples. (A) Paratumor sample of P11; (B) Tumor sample of P11; (C) Paratumor sample of P15; (D) Tumor sample of P15; (E) Paratumor sample of P19; (F) Tumor sample of P19; (G) Paratumor sample of P24; (H) Tumor sample of P24; (I) Paratumor sample of P25; (J) Tumor sample of P25. [file Image2.tif]

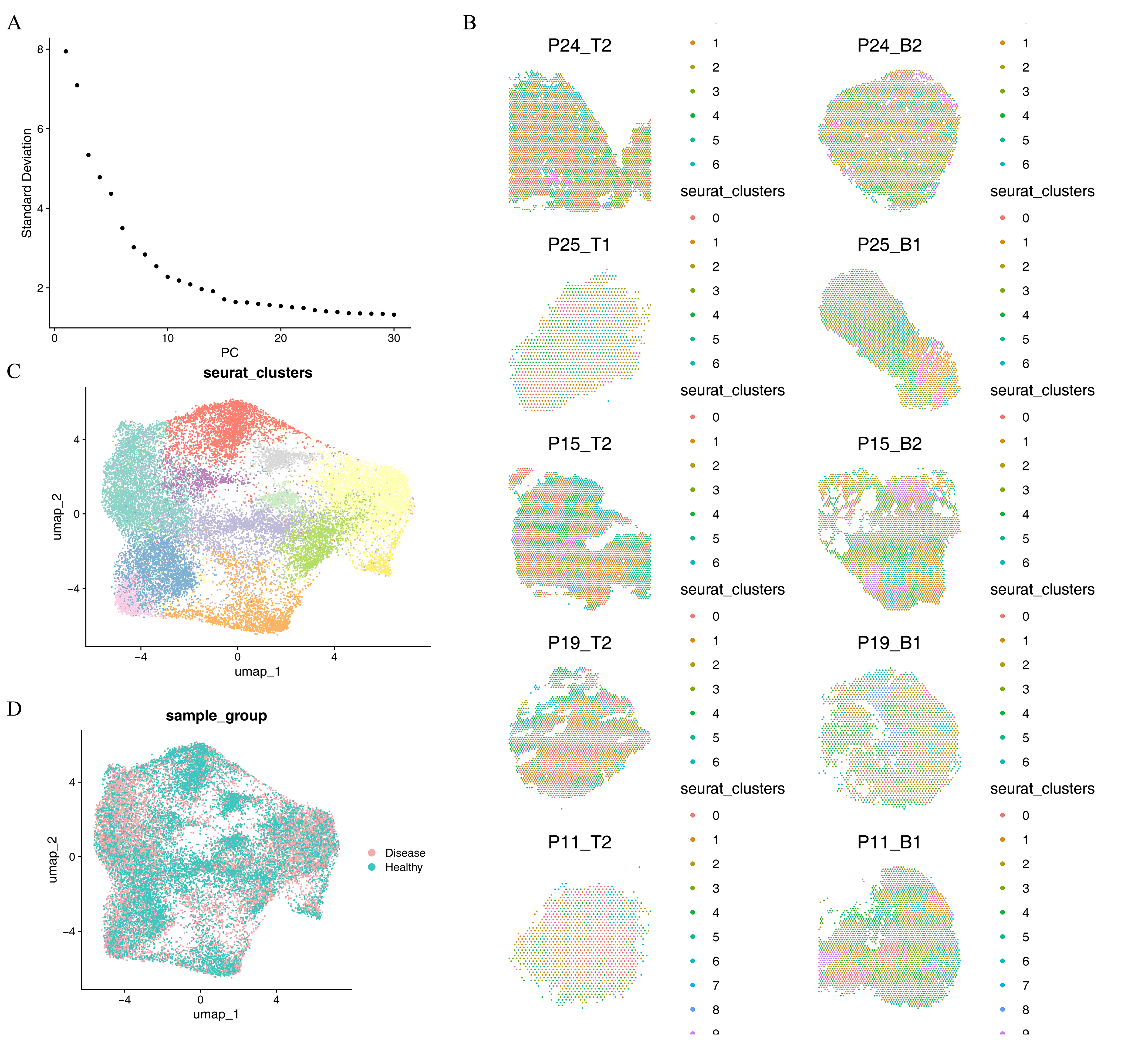

Supplement: Supplementary Figure 3 — Quality control of spatial transcriptomics. (A) Elbow plot; (B) Spatial clustering plots of each paired sample; (C) UMAP dimensionality reduction and clustering plot of all integrated samples; (D) UMAP dimensionality reduction plot colored by sample group (Disease/Healthy). [file Image3.tif]

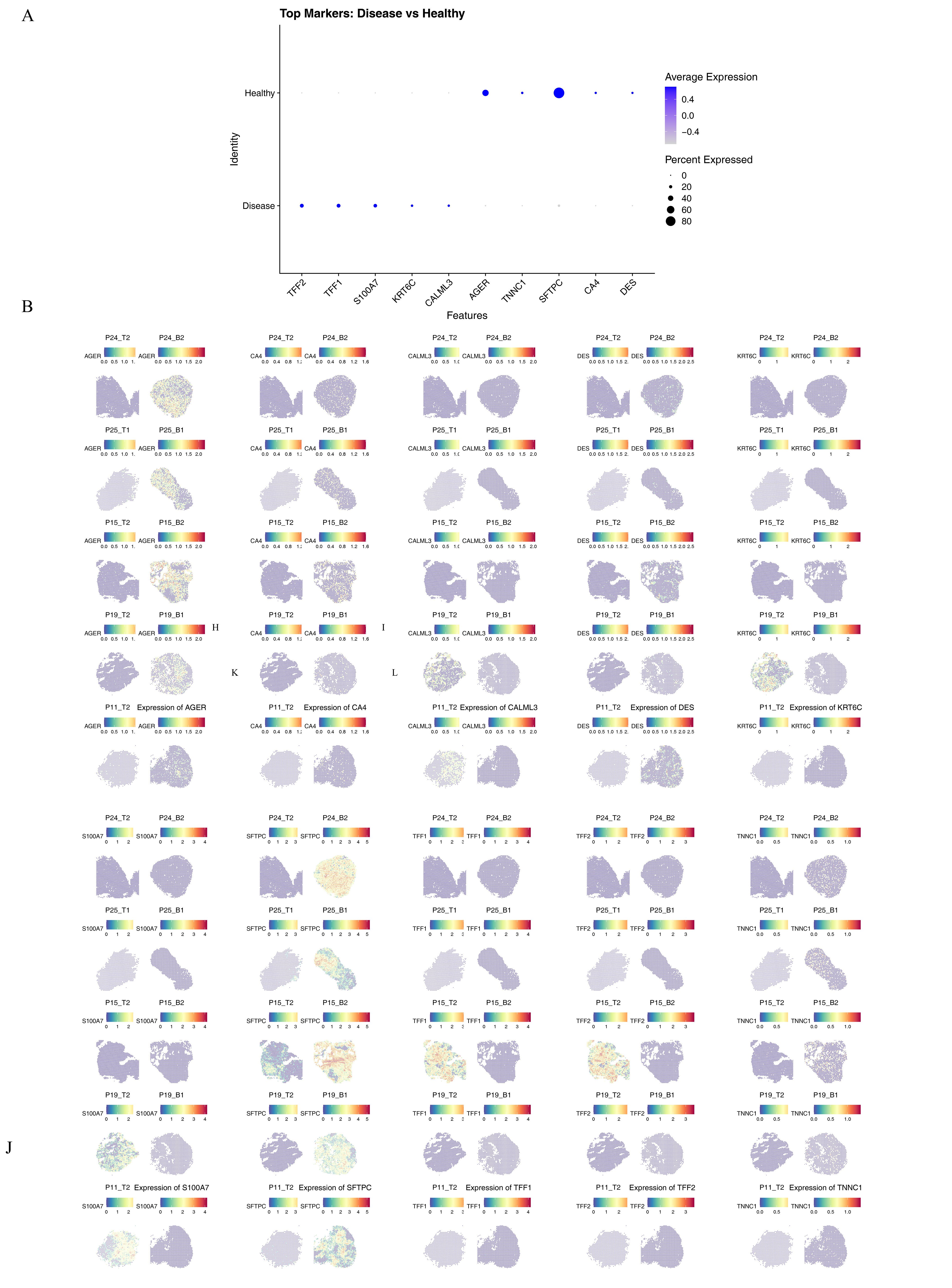

Supplement: Supplementary Figure 4 — Marker gene selection. (A) Expression plots of marker genes; (B) Visualization of spatial expression distribution of marker genes in paired spatial transcriptomic samples. [file Image4.tif]

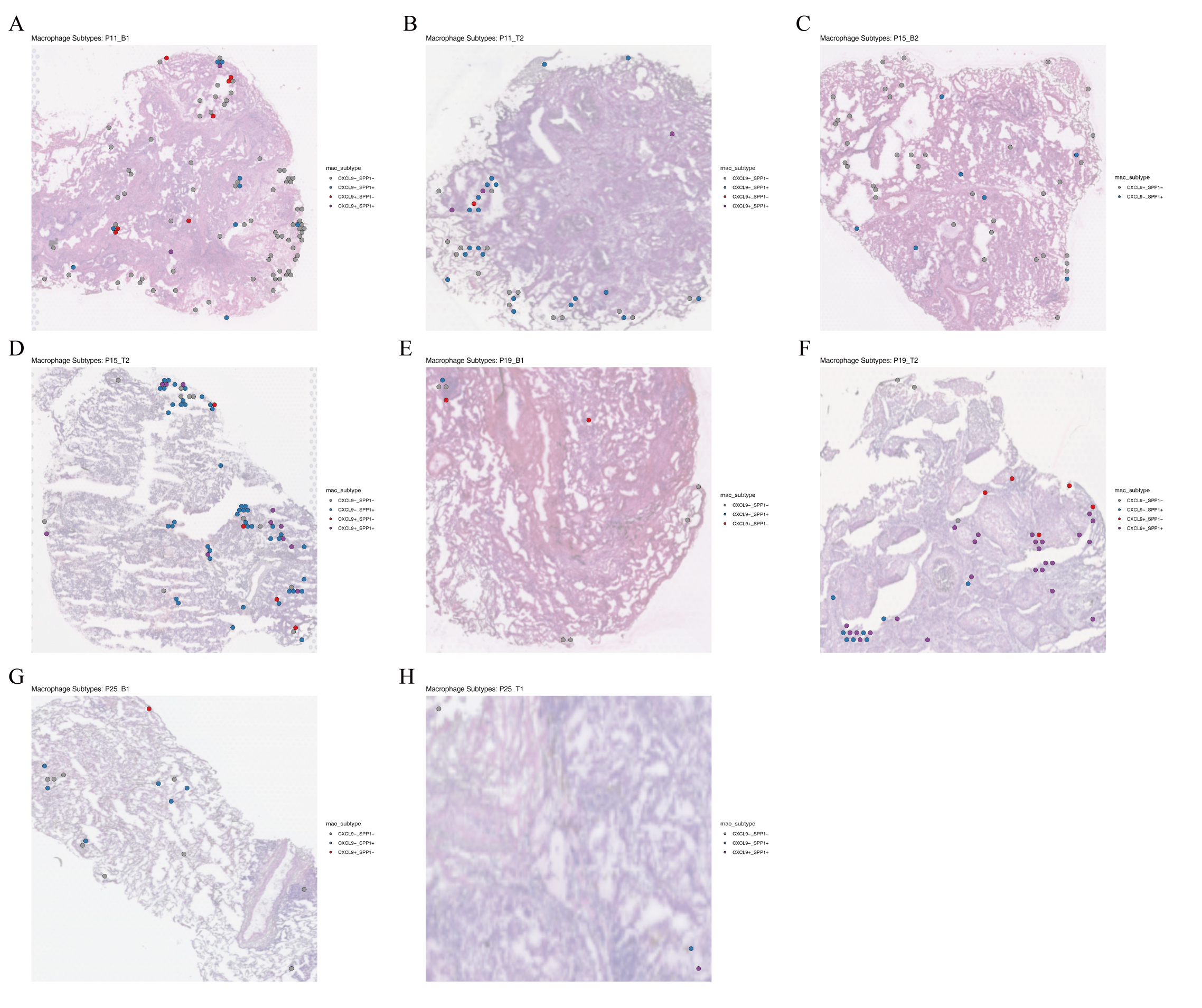

Supplement: Supplementary Figure 5 — Spatial distribution characteristics of macrophage subtypes in paratumor and tumor tissues of NSCLC patients. (A) Paratumor sample of P11; (B) Tumor sample of P11; (C) Paratumor sample of P15; (D) Tumor sample of P15; (E) Paratumor sample of P19;(F) Tumor sample of P19; (G) Paratumor sample of P25; (H) Tumor sample of P25. [file Image5.jpeg]

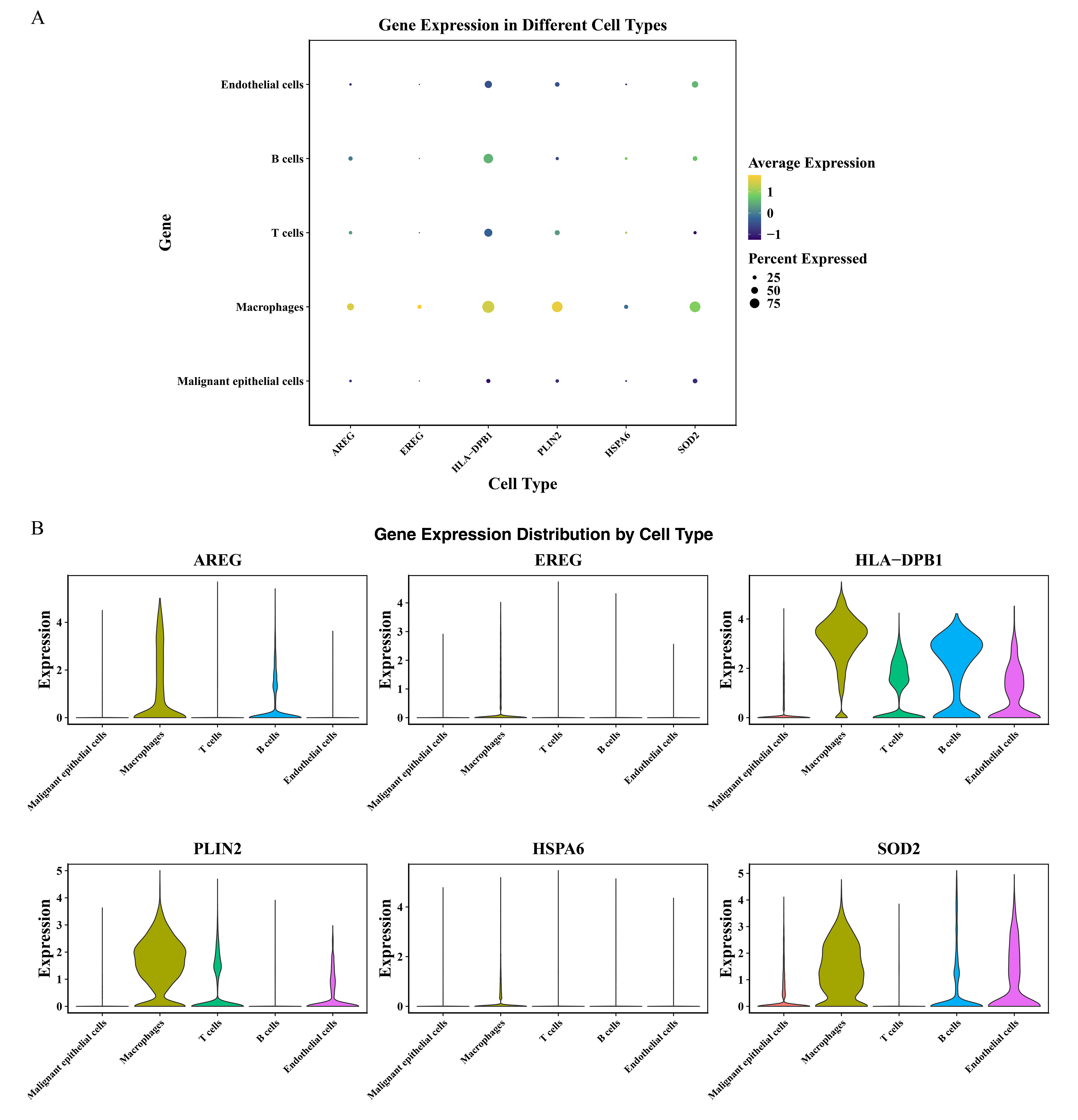

Supplement: Supplementary Figure 6 — Expression of prognostic genes in all cell types. (A) Bubble plot; (B) Violin plot. [file Image6.tif]
