# Supplementary material for: The CXCL9/SPP1 polarity axis in tumor-associated macrophages: immunoregulatory and prognostic significance in non-small cell lung cancer
Source: Front Immunol. 2026 Apr 17;17:1763652. doi: 10.3389/fimmu.2026.1763652 (PMC13132838; doi:10.3389/fimmu.2026.1763652)
Supplement: Supplementary file 7 [file Supplementaryfile1.docx]

# Materials and Methods

**Single-Cell RNA Sequencing Data Analysis**

Detailed quality control and analytical parameters are provided as follows.For the GSE198099 dataset, cells expressing fewer than 200 genes and genes expressed in fewer than 3 cells were first removed. Cells with <200 or >6000 expressed genes, mitochondrial gene proportion >20%, or gene expression count >30,000 were discarded in subsequent filtering steps. Data normalization was performed using the “LogNormalize” and “FindVariableFeatures” functions. The top 2000 most variable genes were selected for PCA, and the optimal number of principal components was determined using an elbow plot. Cell clustering was conducted using the “FindNeighbors” and “FindClusters” functions with a resolution of 0.2.

Functional enrichment analysis of differential cell clusters was conducted using the “analyze_sc_clusters” function in the ReactomeGSA package. The expression distribution of CXCL9 and SPP1 in differential cell populations was visualized. The Wilcoxon rank-sum test was used to analyze the expression differences of CXCL9 and SPP1 between tumor and control groups, with P < 0.05 considered statistically significant.

For macrophage subpopulation analysis, the “FindMarkers” function in Seurat was used with min.pct = 0.25. Thresholds for differentially expressed genes were set as |average log2 fold change| > 0.5 and adjusted P-value < 0.05.

**Spatial transcriptome analysis**

Spatial Transcriptome Data Processing and Integration

Spatial transcriptome data of the samples were read using the Load10X_Spatial function of the Seurat package. Metadata including sample grouping, patient ID, and tissue type were added to each sample; filtering thresholds (nCount>200, nFeature>50) were set based on nCount_Spatial (UMI counts) and nFeature_Spatial (number of detected genes) to complete basic quality control, followed by data normalization which was performed using the SCTransform function.

Subsequently, 3,000 integrated feature genes were screened by the SelectIntegrationFeatures function; after preprocessing via the PrepSCTIntegration function, integration anchors between samples were identified using the FindIntegrationAnchors function, and multi-sample integration was completed with the IntegrateData function. After integration, PCA analysis was conducted based on the integrated assay, and an ElbowPlot was generated to determine the clustering dimensions. Dimensionality reduction and clustering (resolution=0.5) were then completed through the FindNeighbors, RunUMAP, and FindClusters functions.

Differential Marker Gene Screening and Spatial Visualization

Data preprocessing was carried out using the PrepSCTFindMarkers function, followed by screening of differential marker genes between tumor and paracancerous tissues via the FindMarkers function (logfc.threshold=0.25, min.pct=0.1). The top 5 upregulated and downregulated genes were extracted as marker genes, and the spatial expression distribution of these marker genes was plotted using SpatialFeaturePlot.

Cell Type Deconvolution and Key Cell Subtype Classification

Cell type deconvolution analysis was performed using the spacexr package; the spatial distribution of cell types and the proportion of cell types in different groups were visualized, the distribution of cell types was statistically analyzed, and the deconvolution results, statistical tables, and the final annotated Seurat object were saved. Then, the spatial transcriptome objects annotated with cell types by RCTD were loaded and integrated using the Seurat package. Spots annotated as key cells were extracted.

Module scores of key cell subsets were calculated using the AddModuleScore function. Key cells were classified into four subtypes—CXCL9⁺_SPP1⁻, CXCL9⁻_SPP1⁺, CXCL9⁺_SPP1⁺, and CXCL9⁻_SPP1⁻—using expression levels of CXCL9 and SPP1 greater than 0 as the threshold, and the number of each subtype was counted. Statistical analyses were also performed on the quantitative distribution of each subtype across different sample groups.

Neighborhood Analysis and Statistical Testing

Furthermore, the FNN package was loaded to systematically analyze the neighborhood composition ratio of annotated cells and generate raw neighborhood data. Finally, the Wilcoxon test was performed on the neighborhood ratios of key cells using the rstatix and dplyr packages, and paired sample difference analysis was completed according to patient ID and tissue type.

Co-localization and Mutual Exclusivity Analysis

Based on the E-MTAB-13530 dataset, the RCTD-annotated NSCLC spatial transcriptomic Seurat object was read via the Seurat package, the SCT assay was set as the default expression matrix, the expression levels of CXCL9 and SPP1 genes were extracted, and indicator variables for cell types such as is_T_cell and is_Malignant were constructed. High and low expression groups were defined using the 80th and 20th percentiles of CXCL9 and SPP1 expression levels as thresholds, respectively. Fisher’s exact test and Spearman correlation test were adopted to analyze the enrichment differences of cell subsets and the correlation between gene function scores, the overlap coefficient of highly expressed regions of the two genes was calculated to verify spatial exclusivity, and Benjamini-Hochberg FDR correction was performed for p-values derived from non-spatial statistical tests. Subsequently, a k=6 nearest-neighbor weight matrix was constructed based on VisiumV2 spatial coordinates, global and local Moran’s I tests were conducted with the spdep package, and hotspot regions of CXCL9 and SPP1 were identified. Fisher’s exact test and nearest-neighbor distance analysis with 100 permutation tests were combined to verify spatial exclusivity and quantify distance characteristics, accompanied by FDR correction. Finally, parameters including pt.size.factor=2.5 and image.alpha=0.4 were set, and SpatialDimPlot and SpatialFeaturePlot were generated to visualize the spatial distribution, aggregation and exclusivity characteristics of CXCL9 and SPP1, thereby completing the systematic analysis.

**Differential Expression Analysis**

Volcano plots were generated using the R package "ggplot2" to visualize the DEG results. DEGs were ranked by log2FC, and the top 10 upregulated and downregulated genes were labeled in the volcano plots. Heatmaps of the top 10 upregulated and downregulated genes were plotted using the R package "ComplexHeatmap".

**Validation of Prognostic Signatures by Quantitative Real-time PCR**

Primers: AREG (F: 5′-CAGCACCTCTGCATCCAAAC-3′， R: 5′-TGGTACAGGTCCGGTTCTTC-3′)；EREG ( F: 5′-GGCTGTGCTCTATGCCAACT-3′， R: 5′-CCTCCATGGTGTTCCACTTG-3′)；HLA-DPB1 ( F: 5′-GAGAGTGGCGCCTCCGATTA-3′， R: 5′-CCACAGCACAGCAACTCCAG-3′ )；PLIN2 ( F: 5′-GGACCTGGAAGACCTGCTCT-3′， R: 5′-CTCAGCACCTTGGTCACCTT-3′)；HSPA6 ( F: 5′-CCTACACCTTCGGCATCACC-3′， R: 5′-GGTCGTTGATCTTGGCTTCG-3′)；SOD2 ( F: 5′-GGCCAAGGGAGATGTTACAA-3′， R: 5′-GCTTGGCTTCCAGCAACTCT-3′) and GAPDH (F: 5′-GGAGCGAGATCCCTCCAAAAT-3′, R: 5′-GGCTGTTGTCATACTTCTCATGG-3′)；Cxcl9：(F: 5′-GGAGTTCGAGGAACCCTAGTG-3′，R: 5′-GGGATTTGTAGTGGATCGTGC-3′）；Spp1：（F: 5′-CCCAAGCGTGGAAACACACAGCT-3′，R: 5′-TGCCCTTTCCGTTGTTGTCC-3′）；Nos2 (iNOS)：（F: 5′-GTTCTCAGCCCAACAATACAAGA-3′，R: 5′-GTGGACGGGTCGATGTCAC-3′）；Arg1：（F: 5′-CTCCAAGCCAAAGTCCTTAGAG-3′，R: 5′-AGGAGCTGTCATTAGGGACATC-3′）；Gapdh：（F: 5′-AGGTCGGTGTGAACGGATTTG-3′，R: 5′-TGTAGACCATGTAGTTGAGGTCA-3′）.
